# Combined use of principal component analysis/multiple linear regression analysis and artificial neural network to assess the impact of meteorological parameters on fluctuation of selected PM_2.5_-bound elements

**DOI:** 10.1371/journal.pone.0287187

**Published:** 2024-03-20

**Authors:** Siwatt Pongpiachan, Qiyuan Wang, Ronbanchob Apiratikul, Danai Tipmanee, Li Li, Li Xing, Xingli Mao, Guohui Li, Yongming Han, Junji Cao, Vanisa Surapipith, Aekkapol Aekakkararungroj, Saran Poshyachinda

**Affiliations:** 1 NIDA Center for Research & Development of Disaster Prevention & Management, School of Social and Environmental Development, National Institute of Development Administration (NIDA), Bangkok, Thailand; 2 State Key Laboratory of Loess and Quaternary Geology, Institute of Earth Environment, Chinese Academy of Sciences (IEECAS), Xi’an, China; 3 Faculty of Science, Suansunandha Rajabhat, Bangkok, Thailand; 4 Faculty of Technology and Environment, Prince of Songkla University, Phuket, Thailand; 5 School of Geography and Tourism, Shaanxi Normal University, Xi’an, China; 6 National Astronomical Research Institute of Thailand (Public Organization), Chiangmai, Thailand; 7 Asian Disaster Preparedness Center (ADPC), Bangkok, Thailand; Sunway University, MALAYSIA

## Abstract

Based on the data of the State of Global Air (2020), air quality deterioration in Thailand has caused ~32,000 premature deaths, while the World Health Organization evaluated that air pollutants can decrease the life expectancy in the country by two years. PM_2.5_ was collected at three air quality observatory sites in Chiang-Mai, Bangkok, and Phuket, Thailand, from July 2020 to June 2021. The concentrations of 25 elements (Na, Mg, Al, Si, S, Cl, K, Ca, Sc, Ti, V, Cr, Mn, Fe, Co, Ni, Cu, Zn, Ga, As, Se, Br, Sr, Ba, and Pb) were quantitatively characterised using energy-dispersive X-ray fluorescence spectrometry. Potential adverse health impacts of some element exposures from inhaling PM_2.5_ were estimated by employing the hazard quotient and excess lifetime cancer risk. Higher cancer risks were detected in PM_2.5_ samples collected at the sampling site in Bangkok, indicating that vehicle exhaust adversely impacts human health. Principal component analysis suggests that traffic emissions, crustal inputs coupled with maritime aerosols, and construction dust were the three main potential sources of PM_2.5_. Artificial neural networks underlined agricultural waste burning and relative humidity as two major factors controlling the air quality of Thailand.

## Introduction

Over the past few decades, several studies have highlighted the severity of air quality problems in Southeast Asian countries, particularly during the burning seasons [1–4]. Annual agricultural waste burning, which is conducted to prepare the fields for the next crop season, is the major cause of haze in northern, northeastern, and central parts of Thailand between December and April. Open burning is the most economical and convenient land preparation practice for low-income farmers and small agro-industrial enterprises in tropical countries [5–7]. The pre-and post-harvest open burning of sugarcane fields has been acknowledged as one of the main contributors of air pollutants in the northeast and central parts of Thailand [8]. Transboundary haze from peatland fires is also considered a major source of polycyclic aromatic hydrocarbons (PAHs) in PM_0.1_, PM_1_, and PM_2.5_ (particulate matter with aerodynamic diameters less than 0.1, 1, and 2.5 μm) in southern Thailand [9].

Attempts have been made to quantify the potential sources of toxic pollutants, such as PAHs, selected elements, organic carbon (OC), elemental carbon (EC), and water-soluble ionic species (WSIS), using a receptor model with the assistance of principal component analysis (PCA) in the ambient air of Chiang-Mai, Bangkok, and Phuket [1, 3, 10–14]. Although numerous studies have focused on the chemical characterisation of particulate matters, few have reported the levels of some elements in the ultrafine particles in Thailand [12, 14–16].

Three different approaches involving artificial neural networks (ANNs) (i.e. recursive neural network, feedforward neural network (FNN), and multiple linear regression analysis) have been used to forecast and manage air pollutants in several countries [17–19]. A recent study focused compared the ANN model with the backpropagation learning algorithm and Weather Research and Forecasting (WRF) model coupled with Chemistry (WRF-Chem) using the data of various trace gaseous including sulphur dioxide (SO_2_), nitrogen dioxide (NO_2_), ozone (O_3_) and carbon monoxide (CO) [20]. Time-integrated activity modelling, Monte Carlo simulation, ANN modelling, and the combined use of PCA and ANN models were also employed to estimate their capability to evaluate PM_2.5_ contents using 117 older adults with >60 living in Tianjin, northern China [17]. Till date, no study has investigated the impact of meteorological parameters on the alterations of particulate elements using combined PCA and ANNs. The main objectives of this study were to (*i*) chemically characterise particulate elements and other elements in PM_2.5_ collected at Chiang-Mai, Bangkok, and Phuket, (*ii*) apply PCA and ANNs to evaluate the impacts of meteorological parameters on the fluctuation of elemental particulates, and (*iii*) employ risk assessment models to assess the impacts of exposure to airborne elements to human health.

## Material & methodologies

### Air quality observatory sites

**Fig 1:**
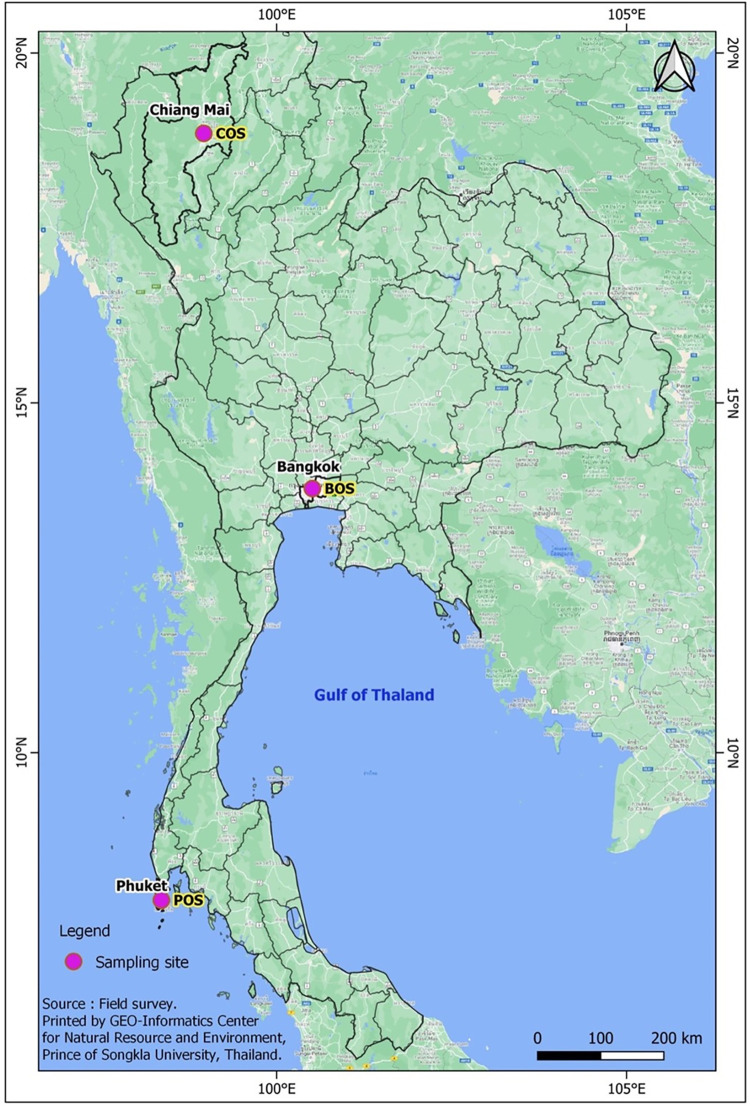
A map of three air quality observatory sites in Thailand.

Three air quality observatory sites were selected in Thailand from north to south: the Chiang-Mai Air Quality Observatory Site (COS), Bangkok Air Quality Observatory Site (BOS), and Phuket Air Quality Observatory Site (POS) (Fig 1). The linear distances between COS and BOS, BOS and POS, and COS and POS were 578, 694, and 1,185 km, respectively. The COS is located at the National Astronomical Research Institute of Thailand (NARIT) on the top of the Doi–Inthanon Mountain, Chiang-Mai Province (18.62994°N, 98.49559°E), which is a part of the Himalaya and is 800–2565 m above sea level. Doi-Inthanon has been recognised as ‘The Roof of Thailand’, covering an area of 482 km^2^. The COS is located on the roofs of the three-storied NARIT office buildings (~10 m above the ground).

In contrast, the BOS is situated in front of the Faculty of Science and Technology, Suan Sunandha Rajabhat University (13.77491°N, 100.50884°E), which is ~1.5 m above the ground level. The university is located in the metropolis of Bangkok (population >10.7 million) and adjacent to numerous tourist attractions, such as the Temple of the Emerald Buddha (Wat Phra Kaew) and Grand Palace. BOS witnesses heavy traffic and is in the vicinity of residential areas and business zones. The POS is situated on the roofs of the three-storeyed buildings (~10 m above the ground) of the Faculty of Technology and Environment, Phuket Campus, Prince of Songkla University (7.89560°N, 98.35214°E). Phuket is Thailand’s largest island, with an area of 576 km^2^, which is slightly less than that of Singapore (729 km^2^). Phuket is situated in the Andaman Sea in southern Thailand, with an N-S trending mountain range in its west.

### Sampling procedures of PM_2.5_

All PM_2.5_ samples were routinely collected in COS (*n* = 82), BOS (*n* = 48), and POS (*n* = 61) using a standard method. A total of 191 sets of PM_2.5_ samples were collected from the three sites from July 2020 to June 2021. It should be noted that emergency measures introduced during the COVID-19 pandemic have resulted in some building access control procedures. For instance, comparatively fewer samples were collected at the BOS owing to strict COVID-19 related lockdown in Bangkok. PM_2.5_ mini-volume samplers (Airmetrics, Springfield, OR, USA) were used to collect aerosol samples at a flow rate of 5 L·min^−1^ in a sampling period of 72 h. Samples were collected on 47-mm diameter polytetrafluoroethylene (Teflon®) membrane filters (PM_2.5_ Air Monitoring PTFE Filters, Whatman Limited, Maidstone, UK) for elemental analysis. Details of the sampling method are provided in the ‘EPA Quality Assurance Guidance Document: Method Compendium, Field Standard Operating Procedures for the PM_2.5_ Performance Evaluation Program, United States Environmental Protection Agency Office of Air Quality Planning and Standards’ [21]. Mass of the PM_2.5_ samples were quantified using the method outlined in the US-EPA Quality Assurance Document: Method Compendium, PM_2.5_, Mass Weighing Laboratory Standard Operating Procedures for the Performance Evaluation Program, United States Environmental Protection Agency Office of Air Quality Planning and Standards [22], and employed microbalances (Mettler Toledo, New Classic MF, MS205DU, Switzerland).

### Analysis of selected elements

The mass of 25 elements (Na, Mg, Al, Si, S, Cl, K, Ca, Sc, Ti, V, Cr, Mn, Fe, Co, Ni, Cu, Zn, Ga, As, Se, Br, Sr, Ba, and Pb) were measured using energy-dispersive X-ray fluorescence (ED-XRF) spectrometry (Epsilon 5 ED-XRF, PANalytical B.V., Netherlands) [23, 24]. A reasonable signal-to-noise ratio was guaranteed using a 3D polarising geometry with 11 secondary targets and one Barkla target. The X-ray beam generated from an X-ray tube with a Gd anode located on the side of the instrument. The accelerating voltage was 25–100 kV and the current was 0.5–24. The maximum power was 600 W. A Ge detector (PAN 32) was used to detect the X-ray radiation. Spectrum of X-ray counts versus the photon energy of each sample was obtained after 30 min of analysis. The energy peaks in the spectrum indicate different elements, and the mass of each element is indicated by its corresponding peak area. The instrument was calibrated using thin-film standards purchased from MicroMatter Co. (Arlington, WA, USA).

### Statistical analysis and chemometrics modelling

#### Principal component analysis (PCA)

PCA is a statistical method that rearranges information into smaller datasets [25]. PCA becomes more powerful when the dataset contains a relatively large number of parameters, such as in spectroscopic data [26]. PCA can explore new parameters or ‘Principal Components (PCs)’, which explain the variability in the dataset. This advanced statistical technique is extremely useful for comprehensively characterising a relatively large dataset with considerably fewer parameters than the original dataset [25]. Hence, PCA has been widely used in the source apportionment of different environmental compartments, such as aerosols [12, 14, 27–31]. In this study, the IBM SPSS Statistics v. 25 combined with a VARIMAX rotation was employed for PCA. The VARIMAX rotation alters the coordinates in the PCA (i.e. orthogonal rotation), which maximises the sum of the variances of the squared loadings. Consequently, all generated coefficients would be either greater than or near zero, with relatively few intermediate values.

#### Artificial neural networks (ANNs)

Sophisticated variants of regression analysis (e.g. multinomial logistic regression, linear mixed models, and nonlinear regression) are seldom insufficient in real-world situations to elucidate the connection between the input parameters and their outcomes. ANNs are mathematical algorithms that construct a massive number of correlated nodes called neurons [32]. ANNs can be used to investigate the interrelationships between dependent and independent parameters, which can identify nonlinear system functions [33]. Generally, ANNs consist of three types of layers: input, output, and hidden. The multilayer perceptron (MLP) is generally recognised as an FNN, which can estimate nonlinear, continuous, and differentiable functions [34, 35]. MLP is applied because of its relatively effective training processes coupled with the need to optimise the weights of the artificial layers of neurons [34, 35].

**Fig 2:**
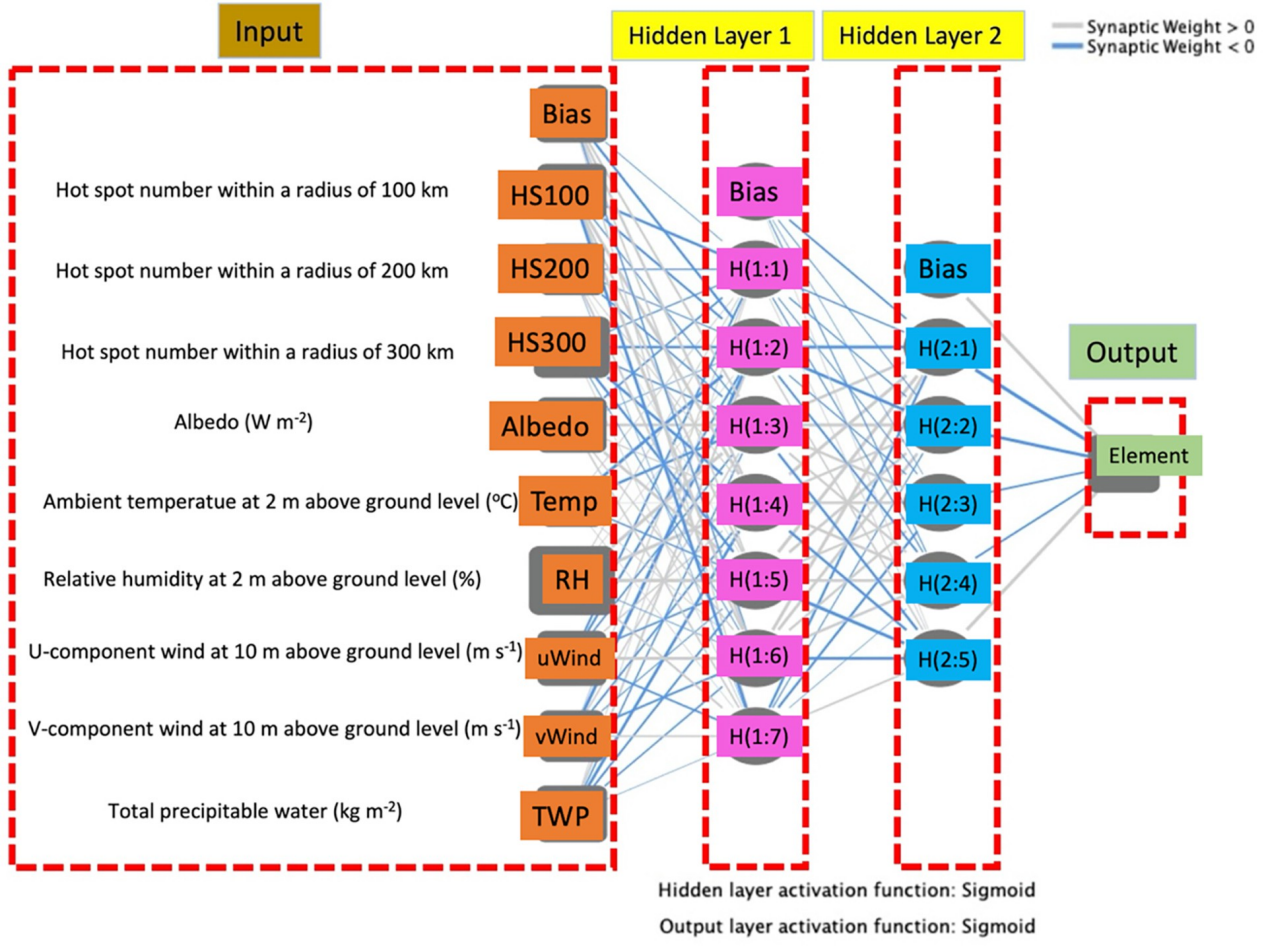
Feed forward neural network for PM_2.5_ bounded element prediction.

Although numerous sources, such as traffic emissions, industrial releases, agricultural waste burning, fireworks, and domestic heating, are responsible for the varying amounts of particulate elements, meteorological parameters (e.g. ambient air temperature (Temp), relative humidity (RH), and wind speed) and hotspots significantly impact the variations of some elements [12, 14–15, 30]. To evaluate this impact on the atmospheric concentrations of the selected 25 elements over a given period, the independent variable importance was calculated using ANNs. The topology of a double-layer neural network with a nonlinear sigmoid transfer function in the two hidden layers and a linear function in the output layer is illustrated in Fig 2. The neurons in the input layer were hotspots within a radius of 100, 200, and 300 km (HS100, HS200, and HS300, respectively), albedo (W m^-2^), Temp at 2 m above ground level (°C), RH at 2 m above ground level (%), *u*-component wind (*u*Wind) at 10 m above ground level (m s^-1^), *v*-component wind (*v*Wind) at 10 m above ground level (m s^-1^), and total precipitable water (TPW) (kg m^-2^). Neurons in the input layer are completely associated with those in the two hidden and the output layers [36]. In addition, hot spot (HS) refers to fire point (i.e. agricultural waste burning), which is the main cause of fire points in Thailand. The above-mentioned parameters (i.e. the input-layer neurons) were selected as the ‘*covariates*’ while atmospheric concentration of each element was selected as the ‘*dependent variable*’. Hypothetically, any mathematical model with a finite number of discontinuities can be estimated by applying a double-layer neural network with a nonlinear sigmoid transfer function in the hidden layer and a linear function in the output layer [37]. Consequently, a double-layer perceptron, which is a class of forward-feed ANNs, was employed in this study. All statistical analyses were performed using the IBM SPSS Statistics v. 25.

#### Back trajectory analysis and meteorological parameters

**Fig 3:**
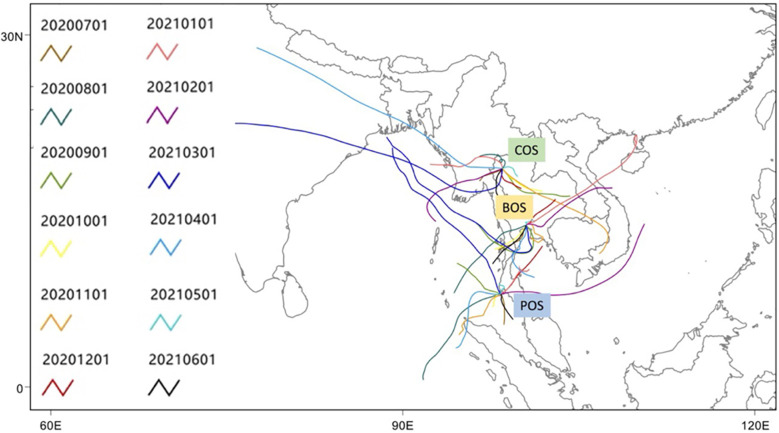
The five-day backward trajectories arriving at the sampling locations named COS (18.59°N, 98.47°E), BOS (13.78°N, 100.51°E), and POS (7.89°N, 98.35°E) at the surface level on the first day of each month from July 2020 to June 2021.

The Hybrid Single-Particle Lagrangian Integrated Trajectory (HYSPLIT, version 4.2.0) model developed by the air resources laboratory of the National Oceanic and Atmospheric Administration (NOAA) [38] was used to calculate the five-day backward trajectories arriving at the surface of the three sites (COS, BOS, and POS) on the first day of each month from July 2020 to June 2021 (Fig 3). Global Data Analysis System meteorological data (1°× 1°) were used as the meteorological input of the HYSPLIT model. Near real-time fire products are created roughly within three hours of satellite observation using the Visible Infrared Imaging Radiometer Suite, which is a hotspot detector provided by the Raytheon Company onboard the polar-orbiting Suomi National Polar-orbiting Partnership and NOAA-20 weather satellites (https://firms.modaps.eosdis.nasa.gov/download/). The goal of the Global Land Data Assimilation System (GLDAS) is to incorporate satellite- and ground-based observational data products using advanced land surface modelling and data assimilation techniques to generate optimal fields of land surface states and fluxes [39]. GLDAS was used to quantify the albedo (reflective quality of the Earth surface) (https://developers.google.com/earth-engine/datasets/catalog/NASA_GLDAS_V021_NOAH_G025_T3H#bands). Other weather conditions were obtained using the Global Forecast System (GFS), a National Centres for Environmental Prediction weather forecast model that produces wind speed, RH, Temp, and soil moisture data. GFS consists of four environmental compartments (air, ocean, terrestrial soil, and sea ice) that work simultaneously to precisely forecast the meteorological conditions (https://developers.google.com/earth-engine/datasets/catalog/NOAA_GFS0P25#bands).

#### Health risk modelling

The mathematical model employed to evaluate the adverse impacts of PM_2.5_-bound elements on human health was originally designed by the US EPA [40]. Over the past few decades, the health risk assessment models of the US EPA have been widely adopted in numerous studies [41–44]. These models can be further classified into non-carcinogenic and carcinogenic impacts [43, 45, 46]. A previous study has highlighted the effects of 30 elements and metalloids (Be, B, Li, Al, Ti, V, Cr, Mn, Co, Ni, Cu, As, Se, Sr, Mo, Pd, Ag, Cd, Sn, Sb, Te, Cs, Ba, W, Pt, Au, Hg, Pb, and Bi) on the human health [47]. Although almost all elements are considered detrimental toxicants, the International Agency for Research on Cancer (IARC) classified five transition metals (As, Cd, Cr, and Ni) as group 1 carcinogens [48]. Pb has been classified in group 2A, indicating lack of evidence on the carcinogenicity of the element in humans with sufficient evidence for experimental animals [49]. The sharing of metabolic pathways between majority of some elements and other essential elements, such as Fe, Zn, Cu, Mn, and Cr, can be due to the identical binding preferences of carcinogenic and essential elements [50]. Furthermore, enzymatic modification is usually not a biochemical process that detoxifies elements. Numerous detoxification mechanisms such as urinary excretion and/or long-term storage (e.g. Cd) exist in nature [51]. One of the main limitations in identifying carcinogenic elements in humans is the lack of large populations with a measurable exposure to a single element [51].

*Health risk assessment for non-carcinogenic elements*. In addition to dermal contact and ingestion, inhalation is another crucial pathway by which humans come in contact with toxic elements that are present in particulate matter. Such exposure is prevalent in people working in the industrial sectors due to the direct emission of air pollutants from on-site facilities and resuspension of dust and particles from the polluted areas. To assess the health risk of non-carcinogenic elements, it is important to calculate the average daily dose (ADD, ng Kg^-1^ day^-1^), which is the product of the atmospheric element concentration (C, ng m^-3^), inhalation rate (IR, m^3^ day^-1^), exposure dose (ED, y), and exposure frequency (EF, day year^-1^) divided by the product of the body weight (BW, kg) and averaging time (AT, d) for non-carcinogens, as illustrated in Eq 1 and S2 Table [52–54].

$$ADD\;(ng\;{Kg}^{-1}{day}^{-1})=\frac{C\times IR\times ED\times EF}{BW\times AT}$$ (1)

Non-carcinogenic impacts can be further assessed by adopting the concept of hazard quotient (HQ), which can be described by Eq 2:

$$HQ=\frac{ADD}{RfD}$$ (2)

where RfD is the reference dose (ng Kg^-1^ day^-1^) (S3 and S4 Tables). The health risk is negligible when HQ of the non-carcinogenic target element is ≤1. In contrast, the negative health impact increases if HQ of the target element is >1 [52, 55, 56].

*Health risk assessment of carcinogenic elements*. Although ingestion and dermal absorption are the two main pathways for pollutants to enter human bodies [45, 57], the extent of the impact of inhalation of carcinogenic elements by humans should also be studied. Since the samples for this study were PM_2.5_, the impact of inhalation of carcinogenic elements would be significant, which is consistent with the US EPA approach. Such impacts are traditionally evaluated by excess lifetime cancer risk (ELCR), inhalation unit risk (IUR), slope factor (SF), and lifetime ADD (LADD, ng kg^-1^ day^-1^). The threshold carcinogenic health risk is stated as 1×10^−6^–1×10^−4^ (EPA, 2005). The relationship between the ELCR and other parameters is presented in Eqs 3 and 4 [58, 59].

ELCR(inhalation)=LADD×SF (3)

$$SF\;=IUR\times \left[\frac{1}{IR}\right]\times BW$$ (4)

Details of the SF and IUR values are provided in S3 Table [58–60]. Despite the numerous parameters (e.g. exposed population, toxicity of pollutants) that influence the potential health risk, the age demographic is an important factor for risk assessment of PM_2.5_-bound carcinogenic elements. WHO defines the ‘Adolescents’, ‘Youth’, and ‘Young People’ as individuals in the age groups of 10–19 years, 15–24 years, and 10–24 years, respectively [61]. However, the age-specific groups employed in the health risk assessment models of carcinogenic elements in this study were adolescents (–12–18 years) and adults (–18–70 years) [52]. Since BOS and POS are located in university campuses and COS is situated at the premises of the NARIT, the selected age groups (adolescents and adults) are appropriate for health risk evaluation.

### Enrichment factors of selected elements in PM_2.5_

Enrichment factors (*EFs*) have been proposed to estimate the impact of traffic emissions, industrial releases, and mining activities on selected atmospheric element concentrations [12, 14, 62–65]. Despite the absence of precise guidelines for categorising the reference element, Al, Si, and Fe, are often used for *EF* calculations [66]. For each selected element, Fe was applied as a reference, assuming small contributions of pollutant Fe and the upper continental crustal composition given by [67]. The *EF* of element *E* in a PM_2.5_ sample can be explained as

$$EF=\frac{{\left(E/R\right)}_{Air}}{{\left(E/R\right)}_{Crust}}$$ (5)

where, *R* is the reference element. If *EF* approaches one, the crust can be considered as the predominant emission source.

## Results & discussion

### Spatial and temporal distribution of PM_2.5_ bounded selected elements

Statistics of the 25 PM_2.5_-bound elements collected at COS, BOS, and POS are illustrated in Table 1. Regardless of the differences in geographical and weather conditions, all three sites showed some similarities among the elemental profiles. First, highest concentrations were observed for Si with average values of 596±45.5, 865±64.7, and 605±43.1 μg m^-3^ at COS, BOS, and POS, respectively. These findings were not surprising because Si is the second most abundant element in Earth’s (continental) crust [68]. Second, Se contents were the lowest with mean values of 3.84±1.28, 16.3±11.7, and 3.63±1.49 ng m^-3^ at COS, BOS, and POS, respectively. This finding is consistent with a previous study of the first National Emission Inventory (NEI) of PM_2.5_ that includes the full suite of particulate trace elements (atomic number > 10) detected at air quality observatory sites across the U.S. [69]. This study reported an exceedingly low emission of Se with the value of 1.4×10^3^ ton yr^-1^ in comparison with those of Si (3.80×10^5^ ton yr^-1^), Al (1.4×10^5^ ton yr^-1^), and Ca (1.3×10^5^ ton yr^-1^) [69]. Relative emission source strength of the 25 elements can be roughly investigated using the ratios of total element concentrations detected at BOS divided by COS and POS. The BOS/COS and BOS/POS ratios for the concentrations of the 25 elements were 1.47 and 1.46, respectively. Accelerated urbanization in Bangkok during the 1980s resulted in rapid increase of personal car ownership and traffic. Consequently, larger emissions of particulate elements occurred, which lead to higher contents in the ambient air, causing the BOS/COS and BOS/POS ratios to be >1. As illustrated in Table 1, the decreasing order of the BOS/COS ratios was Se>Zn>S>Br>As>Pb>K>Fe>Mn>Cu>V>Cr>Ba>Ti>Ga>Cl>Sr>Ca>Na>Mg>Co>Al>Si>Sc>Ni. On the other hand, the descending order of the BOS/POS ratios was S>Br>Zn>K>Se>Pb>As>Ni>Fe>Mn>Cu>Ti>V>Cr>Cl>Ca>Ga>Ba>Sr>Na>Mg>Al>Sc>Co>Si. Since the main contributor of PM_2.5_ in BOS is traffic emissions [10, 12, 70], it appears reasonable to assume that As, Se, Zn, S, Br, and Pb are emitted predominantly from vehicle exhausting sectors. This interpretation is in good agreement with previous studies underlining the importance of fuel combustion as the main sources of As and Se, while Zn and Pb exhaust emissions were dominated by lubricant oil combustion [71]. Furthermore, diesel fuel includes S which primarily derives from the original crude oil source and can still be exist after petroleum refining processes [72]. In addition, the bromine number and bromine index have been extensively studied for evaluating the total reactive olefin content in petrochemicals according to an ASTM procedure [73].

**Table 1:** Statistical description of selected metals in PM_2.5_ collected at COS, BOS, and POS.

		COS (*n* = 82)				BOS (*n* = 48)				POS (*n* = 61)				BOS/COS	BOS/POS
	Unit	Aver	Stdev	Min	Max	Aver	Stdev	Min	Max	Aver	Stdev	Min	Max		
Na	μg m^-3^	17.0	0.930	15.3	19.4	25.6	1.34	20.2	28.1	17.0	0.844	15.5	19.1	1.50	1.51
Mg	μg m^-3^	17.0	0.647	15.8	18.6	25.3	0.941	21.2	27.1	16.9	0.538	15.9	18.2	1.49	1.50
Al	μg m^-3^	64.4	11.2	49.8	91.7	93.7	16.9	60.0	133	63.2	9.11	51.4	87.6	1.45	1.48
Si	μg m^-3^	596	45.5	505	692	865	64.7	611	1003	605	43.1	529	689	1.45	1.43
S	μg m^-3^	1.24	1.22	0.138	7.61	4.69	2.33	0.949	11.9	0.541	0.459	0.112	2.54	3.79	8.67
Cl	μg m^-3^	0.738	0.113	0.549	1.11	1.17	0.310	0.862	2.39	0.671	0.191	0.517	1.65	1.58	1.74
K	μg m^-3^	3.92	2.30	1.86	12.1	9.80	4.37	3.25	19.4	2.09	0.489	1.80	4.73	2.50	4.70
Ca	μg m^-3^	16.4	1.03	14.8	21.1	25.2	1.63	22.3	30.2	15.5	0.285	14.9	16.2	1.53	1.62
Sc	ng m^-3^	251	11.0	225	279	365	21.4	318	409	247	9.05	228	270	1.45	1.48
Ti	ng m^-3^	210	62.2	157	533	354	75.0	270	621	179	15.3	161	244	1.68	1.97
V	ng m^-3^	9.59	2.65	3.21	16.1	18.1	5.92	4.82	33.7	9.79	2.45	3.21	16.1	1.88	1.84
Cr	ng m^-3^	47.2	31.0	25.7	318	84.9	19.9	62.6	164	48.1	7.60	38.5	80.3	1.80	1.77
Mn	ng m^-3^	84.5	27.6	54.6	209	171	41.4	106	332	72.0	16.1	54.6	128	2.02	2.37
Fe	μg m^-3^	1.43	0.634	0.902	4.63	2.95	0.927	1.73	6.71	1.00	0.166	0.809	1.73	2.06	2.96
Co	ng m^-3^	46.2	3.63	38.5	57.8	67.4	6.52	43.4	77.1	46.9	3.49	38.5	54.6	1.46	1.44
Ni	ng m^-3^	85.7	8.56	70.6	116	100	46.1	48.2	193	29.6	5.70	22.5	61.0	1.17	3.38
Cu	ng m^-3^	77.0	95.1	32.1	639	156	133	81.9	925	68.4	45.3	35.3	382	2.02	2.28
Zn	ng m^-3^	226	114	116	665	896	366	332	1941	190	58.7	138	485	3.96	4.71
Ga	ng m^-3^	17.3	2.65	12.8	25.7	27.4	4.57	19.3	38.5	17.2	2.12	12.8	22.5	1.59	1.59
As	ng m^-3^	9.95	5.14	N.D.	22.5	28.4	16.2	N.D.	86.7	8.16	6.95	N.D.	45.0	2.86	3.48
Se	ng m^-3^	3.84	1.28	3.21	6.42	16.3	11.7	4.82	62.6	3.63	1.49	3.21	12.8	4.24	4.48
Br	ng m^-3^	34.9	34.2	3.21	138	104	63.6	14.5	270	14.7	13.8	3.21	89.9	2.98	7.05
Sr	ng m^-3^	135	8.35	116	170	212	16.0	183	260	137	5.23	125	151	1.57	1.55
Ba	ng m^-3^	99.3	12.7	61.0	141	169	16.5	140	217	108	11.2	86.7	148	1.70	1.57
Pb	ng m^-3^	65.8	28.6	28.9	180	165	93.7	62.6	530	41.9	11.6	22.5	109	2.50	3.94

### Enrichment factors (*EFs*) of selected elements

**Fig 4:**
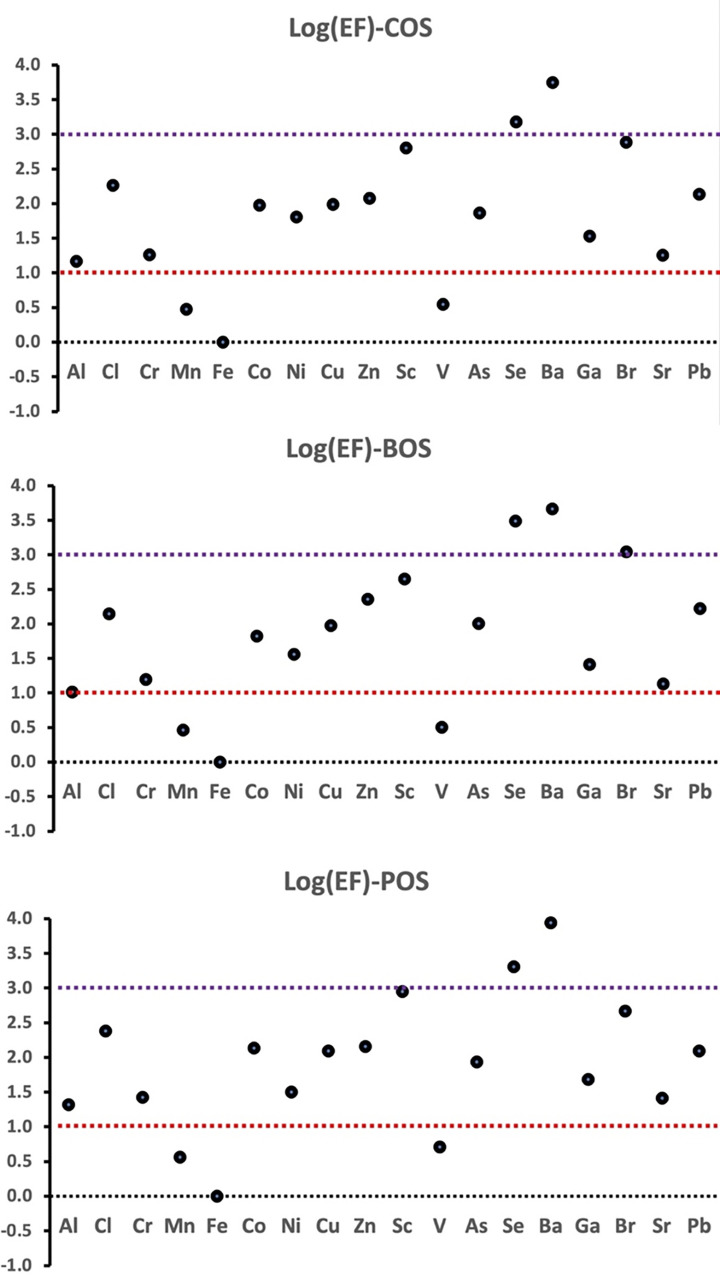
Logarithms of *EF* of 18 selected metals in PM_2.5_ collected at COS, BOS, and POS.

*EFs* of the 18 selected elements are displayed in Fig 4, and the decreasing order of *EFs* in COS and POS was Ba>Se>Sc>Br>Cl>Zn>Co>Pb>Cu>As>Ga>Ni>Cr>Sr>Al>V>Mn>Fe. The sequence of *EFs* in the BOS was Ba>Se>Br>Sc>Zn>Pb>Cl>As>Cu>Co>Ni>Ga>Cr>Sr>Al>V>Mn>Fe. Regardless of some discrepancies between the two sequences, computational results of log(*EF*) can be classified as follows (arbitrary scale) [12, 14, 74]: (*i*) V, Mn, and Fe were not enriched (log(*EF*) <1). (*ii*) Al, Cl, Cr, Co, Ni, Cu, Zn, Sc, As, Ga, Br, Sr, and Pb were slightly enriched (i.e. 1< log(*EF*) <3). (*iii*) Ba and Se were strongly enriched (i.e. log(*EF*) >4). A previous study conducted in Chesapeake Bay, USA, interpreted the majority of atmospheric Al as a consequence of crustal emissions due to its comparatively low *EF* value (i.e. log(EF) <1) [75]. A similar investigation was also performed at seven air quality observatory sites in Bangkok operated by the Pollution Control Department (PCD), Ministry of Natural Resources and Environment, Thailand, which underlined the exceedingly low average log(*EF*) value of Al (-0.44) [14]. These results are consistent with the measured log(*EF*) of Al collected at COS, BOS, and POS, with average values of 1.17, 1.02, and 1.32, respectively (Fig 4). This implies that crustal emissions are likely to be the dominant potential source of Al in the ambient air of Thailand. However, the exceedingly high log(*EF*) values (i.e. >4) of Se and Ba and moderately high log(*EF*) (i.e. 1< log(*EF*) <4) values of Cu and Zn detected at COS, BOS, and POS suggest significant impact of human activities on these two elements, which is probably associated with traffic emissions that is consistent with earlier investigations [76–78]. Several studies have also identified industrial activities as potential emission sources of As, Cr, and Cu [79, 80]. While Sn, Zn, Mo, Sb, Pb, and Cd were deeply associated with industrial activities, high-temperature combustion, and traffic emissions, Ni, Cr, and Cu were interpreted as industrial activity-related elements [81]. Thus, it is concluded that numerous anthropogenic activities, such as vehicular exhaust and industrial activities, are the two main contributors of some elements in PM_2.5_ in the ambient air of Thailand.

### Potential sources of PM_2.5_ bounded elements

PCA can be used to classify the potential contributors of the selected elements from the PM_2.5_ data. PCA applications are based on the assumption that the duration for which a particulate element will remain in the atmosphere of an air quality observatory site (i.e. COS, BOS, and POS) will theoretically be the same for elements from the same contributor. The selected elements were quantitatively characterised in a relatively large number of samples from a single site over the study period. Selected elements of comparable variability were combined together using comparatively fewer factors to account for the inconsistent dataset. Each factor was hypothesised to be deeply connected to the source or source type. Despite the range of potential sources of PM_2.5_, this approach can only distinguish a maximum of eight individual source categories at a time, and ineffective discrimination of similar source types is common [82]. Another limitation of PCA is that large numbers of PM_2.5_ samples and statistically independent sources are required for each major source type [83]. A new study also suggests that the comparability between receptor models (RMs) and chemical transport models (CTMs) differed depending on the potential contributor: vehicle emissions and industrial releases were the two sources successfully predicted by the RMs while the comparatively controversial ones were crustal emissions and traffic road particles [84]. Hence, one should interpret the PCA analysis with great cautions due to the relatively complicated emissions sources of PM_2.5_ in three different cities.

PCA explores a linear combination of parameters (e.g. selected element content in PM_2.5_) to select the greatest variance among them. Furthermore, PCA eliminates this variance and explores a second linear combination that accounts for the largest proportion of the remaining variance. This statistical process is called the principal axis method, and forms orthogonal (uncorrelated) factors. Consequently, the maximum combination that explains the majority of the variance, develops into principal component 1 (PC1), and the second largest combination explains the next largest amount of variance that eventually develops into principal component 2 (PC2), and so on. It is also crucial to underline that some previously reported chemical species (e.g. TC, OC, EC, Cl^-^, NO_2_^-^, NO_3_^-^, SO_4_^2-^, Na^+^, NH_4_^+^, K^+^, Mg^2+^, and Ca^2+^) in PM_2.5_ collected at three sampling sites were also used to enhance the reliability of PCA in this section [85]. The three main principal components (i.e. PC1, PC2, and PC3) of the 25 selected elements coupled with three carbonaceous aerosols and nine water soluble ionic species (WSIS) show interesting findings related to the potential sources of PM_2.5_ in the ambient air of COS, BOS, and POS (Table 2).

**Table 2:** Principal component analysis of selected metals in PM_2.5_ collected at COS, BOS, and POS.

	COS			BOS			POS		
	PC1	PC2	PC3	PC1	PC2	PC3	PC1	PC2	PC3
Na	0.079	**0.961**	0.033	**0.951**	0.174	0.041	0.014	0.000	**0.956**
Mg	0.059	**0.975**	0.041	**0.963**	0.176	0.034	0.017	0.028	**0.973**
Al	0.040	**0.596**	0.264	**0.682**	0.096	-0.196	-0.004	0.257	**0.576**
Si	-0.206	**0.905**	-0.139	**0.935**	0.078	-0.032	-0.282	-0.157	**0.876**
S	**0.879**	0.047	0.195	0.406	**0.655**	0.405	**0.909**	0.158	-0.016
Cl	0.375	-0.247	-0.092	**0.601**	0.225	0.240	0.213	0.269	-0.146
K	**0.933**	0.003	0.114	0.327	**0.870**	0.156	**0.950**	0.192	-0.056
Ca	0.252	-0.021	0.122	**0.927**	0.192	0.247	**0.629**	**0.558**	0.036
Sc	-0.122	0.177	0.017	**0.958**	0.046	0.128	0.018	0.357	0.161
Ti	**0.877**	0.036	0.164	**0.547**	0.118	**0.740**	**0.893**	0.136	0.030
V	-0.055	-0.177	0.126	**0.671**	-0.042	0.226	-0.044	0.260	-0.234
Cr	-0.152	-0.040	-0.079	**0.755**	0.390	0.046	-0.326	-0.292	-0.101
Mn	**0.820**	0.038	0.054	**0.608**	**0.513**	**0.518**	**0.851**	0.034	0.039
Fe	**0.906**	0.011	0.126	**0.509**	0.261	**0.754**	**0.920**	0.153	0.027
Co	-0.110	0.348	-0.073	**0.915**	0.038	0.080	-0.073	-0.203	0.233
Ni	-0.028	-0.018	**-0.805**	0.160	**0.749**	-0.207	0.664	**0.600**	0.133
Cu	0.256	0.009	-0.261	0.259	0.421	-0.126	0.164	-0.132	-0.074
Zn	**0.798**	0.003	-0.047	**0.699**	0.424	0.330	**0.883**	-0.123	-0.054
Ga	0.206	0.180	-0.065	**0.751**	0.181	0.215	0.209	-0.081	-0.155
As	**0.621**	-0.149	-0.104	0.456	**0.735**	0.115	0.462	-0.090	-0.051
Se	**0.624**	-0.027	0.119	**0.509**	0.440	-0.176	**0.631**	-0.073	0.007
Br	**0.940**	-0.011	0.225	0.179	**0.867**	0.170	**0.962**	0.139	-0.095
Sr	**0.579**	0.100	-0.074	**0.936**	0.072	0.250	0.442	-0.040	-0.152
Ba	-0.274	-0.191	**-0.502**	**0.912**	0.062	0.075	-0.459	-0.474	-0.181
Pb	**0.840**	-0.007	-0.042	0.415	**0.698**	0.256	**0.922**	0.113	0.016
TC	**0.890**	-0.041	0.088	-0.069	**0.943**	0.042	**0.906**	0.228	-0.080
OC	**0.874**	-0.038	0.098	-0.091	**0.931**	0.004	**0.897**	0.237	-0.086
EC	**0.911**	-0.047	0.059	-0.009	**0.922**	0.135	**0.914**	0.199	-0.064
Cl^-^	-0.199	-0.133	0.413	0.236	-0.033	-0.163	-0.164	**0.640**	-0.114
NO_2_^-^	0.401	0.244	0.006	0.117	0.271	-0.118	0.193	0.084	0.119
NO_3_^-^	**0.849**	-0.067	0.238	0.462	**0.690**	0.119	**0.895**	0.297	-0.016
SO_4_^2-^	**0.891**	-0.022	0.314	0.075	**0.601**	**0.657**	**0.926**	0.232	-0.015
Na^+^	0.382	-0.012	**0.707**	0.425	0.168	0.104	**0.624**	**0.632**	0.123
NH_4_^+^	**0.913**	-0.049	0.193	-0.023	**0.770**	**0.502**	**0.936**	0.111	-0.036
K^+^	**0.941**	-0.055	0.145	0.077	**0.890**	0.129	**0.954**	0.228	-0.069
Mg^2+^	0.106	-0.044	**0.835**	-0.417	-0.014	0.123	0.366	**0.864**	0.023
Ca^2+^	0.415	-0.022	**0.675**	-0.392	0.122	0.456	**0.625**	**0.738**	0.011
% of Total Variance	39.4	9.85	8.67	45.3	18.3	7.07	46.5	9.65	7.22

Please note that any value higher than 0.5 was highlighted in bold font

First, considerably strong positive correlations of Pb, Zn, Br, TC, OC, EC, NO_3_^-^, SO_4_^2-^, and S were observed in the PC1 of COS and POS. Based on a PCA-based receptor model analysis, vehicular exhaust was found to be the main contributor of Pb in the PM_2.5_ samples that were collected at two monitoring sites in the Athens Basin: Patission Street in Athens City Center and Rentis, a semi-urban industrial area, between March 1995 and March 1996 [86]. Although Pb has been widely used as a geochemical tracer to identify coal-based heating activities in different cities [87–88], it can also be used as an indicator of traffic emissions due to the comparatively warm climate conditions of Thailand, coal-based heating stoves should be considered only a minor source. Furthermore, particles from fossil fuel combustion also involve various types of sulphur that form acidic sulphur compounds in the aerosols (e.g. (NH_4_)_2_SO_4_, (NH_4_)HSO_4_, H_2_SO_4_) [89]. Previous source apportionment studies have also concluded that trace amounts of Zn and Br are emitted from vehicle exhausts [90–92]. An earlier study also highlighted the effects of day-of-week trends and vehicle types on PM_2.5_-bound TC, OC, and EC collected at the city centre of Bangkok [70]. Additionally, the impacts of vehicle exhausts on atmospheric particulate NO_3_^-^ were comprehensively reported in ambient air of numerous cities in China [93–95]. It is also important to underline that the strong positive correlation coefficients of K^+^ can be interpreted as a consequence of wood smokes and agricultural waste combustions [96]. Hence, PC1 was inferred as a mixture of ‘traffic emissions’ and ‘biomass burnings’, and contributed to 39.4 and 46.5% of PM_2.5_ at COS and POS, respectively. These findings are consistent with earlier studies using PAHs, OC, EC, and WSIS for source characterisation of particulate matter collected at COS, BOS, and POS, indicating that vehicle exhaust and biomass burning are two main contributors of PM_2.5_ in the ambient air of the respective cities [1, 10–11].

Second, the moderately strong positive correlation coefficients (i.e. >0.5) of Al, Na, Mg, and Si were observed in PC2, PC1, and PC3 of COS, BOS, and POS, respectively. A study on the size-based chemical characterisation of PM_2.5_ at a roadside environment in Beijing, China, showed that Al, Si, and Mg were strongly associated with the resuspended road dust [97]. Natural crustal emissions are responsible for the comparatively high proportions of Ca, Al, Si, Fe, and K [98]. A recent study using transmission electron microscopy with ED-XRF spectrometry revealed that sea salt aerosols (SSAs) collected at Ny-Ålesund, Svalbard, during summer contained cubic NaCl coated with a mixture of NaNO_3_, Na_2_SO_4_, Mg(NO_3_)_2_, and MgSO_4_ [99]. Thus, it can be inferred that SSA contains comparatively high amounts of Na, Mg, S, and Cl. After considering the potential sources of Na, Mg, and Si, it is suggested that these PCs are a mixture of crustal particles (i.e. resuspended road dusts) and maritime aerosols, which contribute 8.67, 45.3, and 9.65% of PM_2.5_ in COS, BOS, and POS, respectively. The origin of some of the five-day backward trajectories in the Andaman Sea supports this interpretation (Fig 3).

Additionally, the relatively high correlation coefficients of Ca, Sc, Ti, Mn, Fe, and Sr were also detected in PC1 of BOS. Urbanisation in Thailand has mainly occurred in the Bangkok metropolitan area, which increased from 1,900 to 2,100 km^2^ between 2000 and 2010, making it the fifth-largest metropolitan in East Asia, and putting it above megacities, such as Jakarta, Manila, and Seoul. Consequently, numerous ongoing construction projects (e.g. Bangkok Mass Transit System, Metropolitan Rapid Transit, and One Bangkok (the new vertiginous real estate project of Bangkok)) have been accused of releasing pollutants in the ambient air of Bangkok over the past few years. Chemical fingerprints in urban fugitive PM_2.5_ samples collected from 11 northern Chinese cities indicated that Ca, Si, Fe, and Al were the most abundant, with Ca having the highest concentration among the 22 measured elements in construction dust samples [100]. Thus, it was inferred that construction dusts coupled with resuspended road particles accounted for ~45.3% of PM_2.5_ in the ambient air in Bangkok.

**Fig 5:**
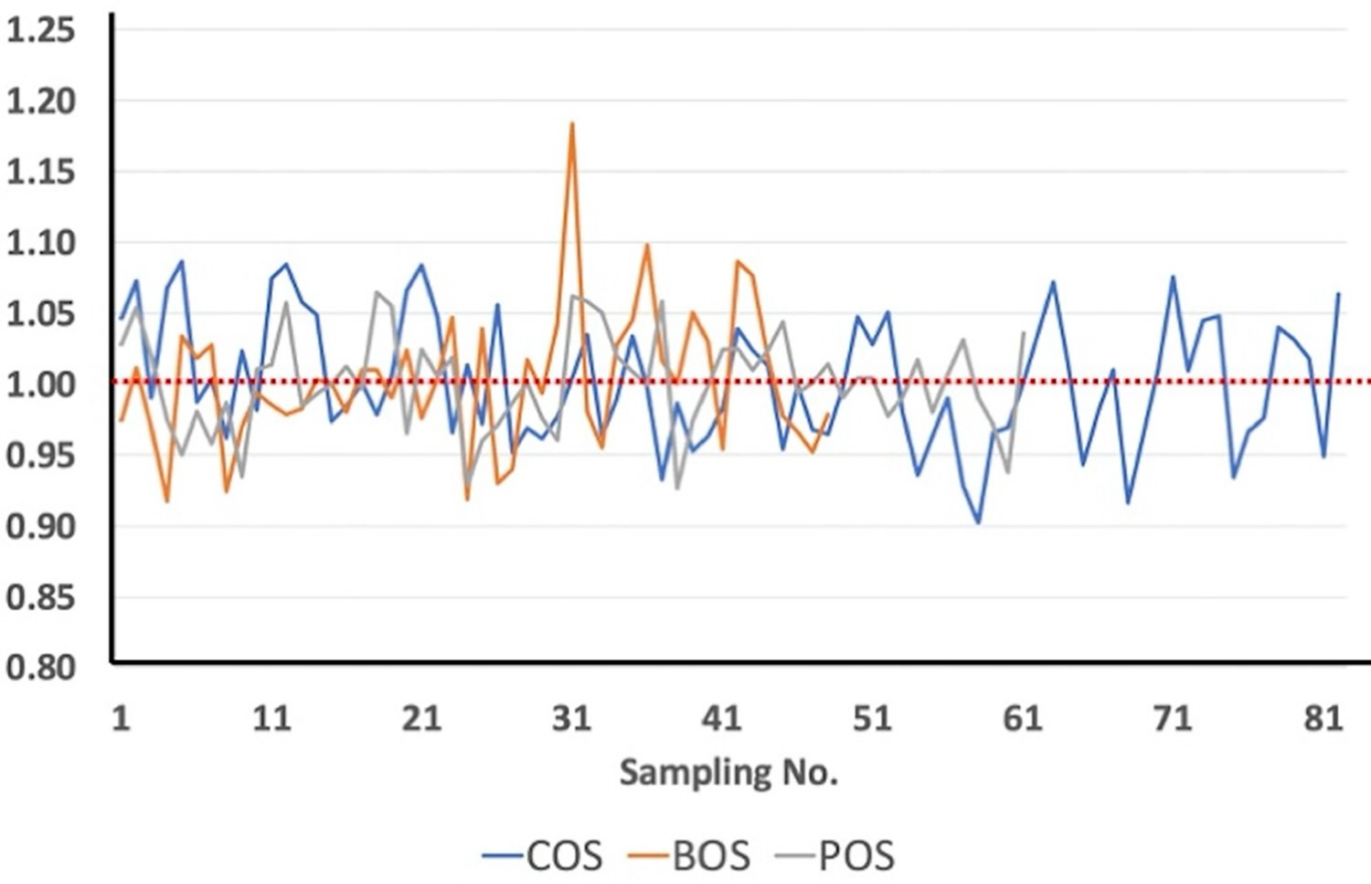
Binary ratios of sum of PM_2.5_ bounded 25 elements as predicted by PCA-MLR to those of measured values (M/P) at COS, BOS and POS.

In spite of the fact that PCA applied to the data set gives qualitative information related to the contributors of the air pollutants, it is not sufficient enough for providing quantitative insights associating with the contributions of each source category. To combat these challenges, the multiple regression algorithm was employed to the data set following the PCA (i.e. PCA-MLRA: Principal Component Analysis with Multilinear Regression Receptor Modelling) [101–104]. In this study, PCA and PCA-MLRA were employed to classify the potential sources of PM_2.5_ in ambient air of three air quality observatory sites. The accuracy of the PCA-MLRA algorithm can be tested by computing “measured to predicted” ratios of the air pollutants, since PCA-MLRA provides the contribution of each contributor of the content of each chemical compound in the identical sample [105]. After varimax rotation, three principal components (i.e. eigenvalue greater than one) were extracted by PCA as displayed in Table 2. The contributions of these three PCs were further evaluated by PCA-MLRA model as illustrated in S1 Table. The measured to predicted ratios (M/P) of the selected elements varied between 0.90 (17/02/2021; COS) to 1.18 (27/03/2021; BOS). It is obviously displayed in S1 Table that these 25 selected elements are estimated with an uncertainty of better than 5%. Overall, the average values of M/P of 25 selected elements for each sampling site were close to one as displayed in Fig 5. According to the M/P ratios illustrated in Fig 5, the fitted results in this study can be accepted.

### Binary ratios of selected elements

According to the CRC Handbook of Chemistry and Physics, the sequence of abundance of chemical elements in Earth’s (continental) crust is O>Si>Al>Fe>Ca>Na>Mg, contributing 46.1, 28.2, 8.23, 5.63, 4.15, 2.36, and 2.33%, respectively [68]. Numerous ratios of selected elements have been used to quantify the crustal emissions, biomass burning, vehicular exhaust, industrial release, and shipping activities [15, 27, 97, 100]. The impact of geographical conditions combined with seasonal constraints on the variations in Ca/Al, Ca/Si, Zn/Al, Pb/Al, Fe/Al, Na/Mg, Ni/V, V/Fe, As/V, and Se/V were investigated using analysis of variance (ANOVA) and *t*-Test (Table 3). Since Al and Fe are the third and fourth most abundant elements in the Earth’s crust, Fe/Al ratio can be used to assess the crustal input in PM_2.5_ at COS, BOS, and POS. The Fe/Al ratio of the Earth’s crust is 0.68 [68] or 0.33 [67]., which are much higher than those detected at the three sites, indicating that crustal emissions are not the only potential source of PM_2.5_ in the ambient air of Thailand. Non-seasonal significances of Ca/Al and Ca/Si were also observed at all monitoring sites. Previous studies have employed Ca as a geochemical tracer of construction dust [97, 106]. Significant reductions in Ca/Al and Ca/Si during the Beijing Olympics were consistent with the fact that all construction sites were temporarily shut down to improve air quality [106]. Since there were no major events that were crucial enough to cease all construction projects in Thailand during the monitoring period, we infer the non-seasonal significance of Ca/Al and Ca/Si as a consequence of regular emissions of construction dust from associated sites adjacent to all three study sites. Although Na and Mg have been widely applied as indicators of SSAs [15, 99, 107], Na/Mg ratios must be interpreted carefully. All Na/Mg ratios detected in COS, BOS, and POS were close to 1.00, which is consistent with the Na/Mg ratio (1.01) reported in the CRC Handbook of Chemistry and Physics [68]. It is noteworthy that the Na/Mg ratios obtained in this study were approximately eight times lower than those of sea-spray aerosols [15]. Moreover, the Pearson correlation coefficients of Na and Mg from the three monitoring sites were exceedingly high (*r*^2^ >0.9), indicating a similar potential source (S5–S7 Tables). Thus, crustal emissions visibly influence the variations of Na and Mg in PM_2.5_, regardless of discrepancies observed with the application of Fe/Al.

**Table 3:** Statistical descriptions of selected elemental ratios in PM_2.5_ collected at COS, BOS, and POS.

Site	Season		Ca/Al	Ca/Si	Zn/Al	Pb/Al	Fe/Al	Na/Mg	Ni/V	V/Fe	As/V	Se/V
		*n*	Song et al., 2012		Shen et al., 2016		Kayee et al., 2020		Pongpiachan et al., 2021			
COS	March-June	22	0.252±0.0511	0.0287±0.00322	0.00416±0.00250	0.00121±0.000660	0.0291±0.0179	1.01±0.0193	7.95±2.42	0.00651±0.00326	1.07±0.646	0.498±0.283
	July-October	31	0.271±0.0407	0.0274±0.00269	0.00312±0.00191	0.000774±0.000199	0.0181±0.00448	1.00±0.0189	10.1±2.86	0.00902±0.00263	0.809±0.480	0.366±0.100
	November-Februay	29	0.261±0.0481	0.0274±0.00287	0.00366±0.00424	0.00125±0.000914	0.0227±0.0129	1.00±0.0176	10.8±4.95	0.00674±0.00279	1.54±1.65	0.494±1.10
	ANOVA (*p*<0.05) *F*_Critic_* = 3.109		*F* = 1.103	*F* = 1.615	*F* = 0.751	*F* = 4.776	*F* = 5.129	*F* = 2.337	*F* = 4.009	*F* = 6.687	*F* = 3.500	*F* = 0.358
	Statistical Significance		**NS**	**NS**	**NS**	**S**	**S**	**NS**	**S**	**S**	**S**	**NS**
BOS	March-June	24	0.281±0.0493	0.0292±0.00302	0.00928±0.00433	0.00131±0.000533	0.0339±0.0135	1.01±0.0199	3.23±0.838	0.00723±0.00270	0.982±0.444	0.594±0.634
	July-October		N/A	N/A	N/A	N/A	N/A	N/A	N/A	N/A	N/A	N/A
	November-Februay	24	0.273±0.0491	0.0293±0.00231	0.0105±0.00439	0.00216±0.00118	0.0316±0.0126	1.01±0.0165	10.6±6.76	0.00585±0.00235	2.88±2.34	1.72±1.61
	*t*-Test (*p*<0.05) Critical Value = 1.678		*t*-differ** = 0.563	*t*-differ = -0.129	*t*-differ = -0.969	*t*-differ = -3.216	*t*-differ = 0.61	*t*-differ = 0.00	*t*-differ = -5.3	*t*-differ = 1.889	*t*-differ = -3.904	*t*-differ = -3.188
	Statistical Significance		**NS**	**NS**	**NS**	**S**	**NS**	**NS**	**S**	**S**	**S**	**S**
POS	March-June	30	0.242±0.0329	0.0256±0.00186	0.00279±0.00090	0.000611±0.000150	0.0148±0.00283	1.00±0.0190	3.11±1.13	0.0105±0.00254	0.787±0.568	0.365±0.151
	July-October	13	0.260±0.0282	0.0259±0.00183	0.00322±0.00063	0.000695±0.000142	0.0169±0.00275	1.01±0.0212	2.99±0.647	0.0106±0.00195	0.853±0.828	0.353±0.0910
	November-Februay	18	0.257±0.0336	0.0261±0.00214	0.00347±0.00159	0.000799±0.000349	0.0178±0.00454	1.00±0.0205	3.74±1.79	0.00875±0.00290	1.03±0.804	0.495±0.389
	ANOVA (*p*<0.05) *F*_Critic_ = 3.150		*F* = 1.976	*F* = 0.391	*F* = 2.245	*F* = 3.905	*F* = 4.779	*F* = 1.289	*F* = 1.732	*F* = 3.111	*F* = 0.681	*F* = 1.983
	Statistical Significance		**NS**	**NS**	**NS**	**S**	**S**	**NS**	**NS**	**NS**	**NS**	**NS**

*F_Critc_ stands for *F* critical value at *p*<0.05

**t-differ stands for *t*-difference at *p*<0.05

Influence of industrial release and shipping activities on the fluctuation of some selected elements in PM_2.5_ was further studied by examining the Ni/V, V/Fe, As/V, and Se/V ratios (Table 3). The Ni/V, V/Fe, As/V, and Se/V ratios at the Laem Chabang Sea Port (LCSP), which is the main deep-sea port of Thailand, were reported as 1.6, 0.005, 10.4, and 0.51, respectively [27]. Ni/V and As/V ratios detected at the LCSP were significantly different from the values obtained in this study. This indicates that industrial exhaust and shipping emissions have negligible effects on PM_2.5_ compositions in the three studied cities of Thailand. Significant periodic differences in the Ni/V, V/Fe, and As/V ratios in PM_2.5_, at COS and BOS, involve the effects of seasonal cycle of air pollution in the northern and central parts of Thailand. Forest fires and agricultural waste burnings are one of the main contributors of the deteriorating air quality over the past two years, particularly in the northern Thailand [1, 70, 108]. Pre-harvest and post-harvest open burnings in sugarcane fields have been recognised as the main sources of air pollutants in central Thailand [8]. Because the harvesting cost required for hiring cutting machines is relatively expensive, small-scale sugarcane farmers have little choice but to burn the leftover sugarcane because of the ease of finding labour [109]. Additionally, wood combustion and sugarcane burning have been reported as potential contributors of As, Ni, Se, and V in particulate matter emitted from cogeneration power plants and factories that use wood boilers [110–112]. Thus, it is inferred that the significant differences in Ni/V, V/Fe, and As/V ratios of PM_2.5_ samples collected at COS and BOS during haze area consequence of agricultural waste burning which usually starts from January to May (Table 3 and S1 Fig). According to the annual report of Geo-Informatics and Space Technology Development Agency from 2020 to 2021, the total hot spot numbers (i.e. the sum of hotspots numbers detected in Thailand, Vietnam, Myanmar, Lao PDR, and Cambodia) showed the highest value in March (61717 in 2020; 64,760 in 2021), followed by February (38,218 in 2020; 31,174 in 2021) and April (30,006 in 2020; 29,907 in 2021) (see S1 Fig). Overall, these findings have shown that policymakers should be aware that the expansion of the sugarcane industry can inevitably lead to seasonal air quality problems, and thus non-burning alternative agricultural practices are essential.

### ANNs of elements in PM_2.5_

To enhance the reliability of the simulations and generate better predictions, ~70% of the measured data was used for training, and the remaining 30% was applied for validation (Table 4). Several simulations were repeatedly conducted to determine the best combination of the hidden layers and their neurons, sorting algorithm, and optimum learning speed. Nine covariates, namely HS100, HS200, HS300, albedo, Temp, RH, *u*Wind, *v*Wind, and TPW, were standardised using the rescaling method. Two hidden layers were processed with a sigmoid activation function. The output layer was PM_2.5_ bounded concentrations of 25 elements, which were normalised by rescaling with a sigmoid activation function. Some squared errors (SSEs) were selected as error functions during the training of the basic MLP neural network (Table 4). SSEs make the error function in the ANNs differentiable and square the inaccuracies; thus, they can be applied to decrease the degrees of both positive and negative inaccuracies. Furthermore, SSEs can be used as statistical techniques to investigate the prediction accuracy of ANN models. The training and testing performance of the ANN model can also be evaluated by analysing the training and testing relative errors (Table 4). The percentage contributions of the importance of the independent variables signify the influence of each parameter on the variations in the atmospheric concentrations of the 25 elements. RH is the most influential meteorological parameter governing the fluctuation of atmospheric contents of Na, Mg, Si, S, Cl, K, Ca, Se, Ti, Cr, Mn, Fe, Co, Ni, Ga, As, Se, Br, Sr, and Pb. The hotspot numbers (i.e. HS100, HS200, HS300) also play a crucial role for the atmospheric Cu and Zn concentrations. Hotspot numbers were the second largest covariates controlling the variation in atmospheric concentrations of Na, Mg, Si, S, K, Ti, Mn, Co, Br, and Sr, with percentage contributions of 65.2, 72.2, 82.1, 43.5, 78.0, 89.7, 79.8, 50.6, 46.9, and 85.9%, respectively.

**Table 4:** The case processing summary and independent variable importance using artificial neural network.

	Na	Mg	Al	Si	S	Cl	K	Ca	Sc	Ti	V	Cr	Mn
Case Processing Summary	%	%	%	%	%	%	%	%	%	%	%	%	%
ANNs-Training	63.9	73.8	70.2	73.8	68.1	67.5	73.8	71.7	63.9	66.5	71.2	73.8	72.8
Measured Data-Testing	36.1	26.2	29.8	26.2	31.9	32.5	26.2	28.3	36.1	33.5	28.8	26.2	27.2
Valid	100.0	100.0	100.0	100.0	100.0	100.0	100.0	100.0	100.0	100.0	100.0	100.0	100.0
Model Summary													
Training-Sum of Squares Error	0.886	0.635	2.084	1.051	0.734	0.970	0.347	0.701	0.655	0.700	1.124	0.670	0.996
Training-Relative Error	0.182	0.096	0.669	0.240	0.320	0.590	0.090	0.139	0.158	0.261	0.544	0.857	0.233
Tesing-Sum of Squares Error	0.512	0.349	1.319	0.403	0.270	0.300	0.252	0.167	0.491	0.293	0.320	0.057	0.726
Testing-Relative Error	0.144	0.101	0.485	0.197	0.288	0.642	0.237	0.087	0.149	0.356	0.485	0.670	0.394
Independent Variable Importance	%	%	%	%	%	%	%	%	%	%	%	%	%
HS-100	40.0	40.6	28.1	47.8	22.8	23.0	25.7	35.8	37.6	31.7	39.0	45.0	29.8
HS-200	24.3	34.3	23.5	24.1	10.0	29.0	78.0	29.5	25.5	24.3	15.8	35.6	10.7
HS-300	65.2	72.2	33.9	82.1	43.5	27.1	44.0	18.3	26.5	89.7	12.2	30.8	79.8
Albedo	41.1	39.0	39.3	48.2	27.7	26.5	36.1	42.6	22.9	53.3	20.1	30.3	42.4
Temp	9.1	18.9	86.2	33.4	42.3	48.3	14.1	30.6	10.5	55.1	73.6	71.8	56.8
RH	**100.0**	**100.0**	93.6	**100.0**	**100.0**	**100.0**	**100.0**	**100.0**	**100.0**	**100.0**	37.3	**100.0**	**100.0**
*u*-Wind	39.1	38.3	18.5	48.3	21.7	13.4	28.8	27.4	24.6	2.1	14.2	45.2	34.2
*v*-Wind	22.6	21.9	**100.0**	48.0	18.6	27.4	23.5	18.1	48.9	11.5	**100.0**	99.7	18.8
TPW	45.3	53.2	20.3	53.4	6.1	12.4	9.8	21.8	46.6	25.6	10.9	42.9	6.1
	Fe	Co	Ni	Cu	Zn	Ga	As	Se	Br	Sr	Ba	Pb	
Case Processing Summary	%	%	%	%	%	%	%	%	%	%	%	%	
ANNs-Training	70.7	69.6	65.4	63.4	66.5	72.3	63.9	69.6	66.5	73.3	69.1	69.6	
Measured Data-Testing	29.3	30.4	34.6	36.6	33.5	27.7	36.1	30.4	33.5	26.7	30.9	30.4	
Valid	100.0	100.0	100.0	100.0	100.0	100.0	100.0	100.0	100.0	100.0	100.0	100.0	
Model Summary													
Training-Sum of Squares Error	0.703	0.816	0.338	0.871	0.650	1.494	0.893	1.252	0.649	0.598	1.264	0.562	
Training-Relative Error	0.349	0.160	0.104	0.915	0.266	0.380	0.390	0.548	0.205	0.151	0.442	0.427	
Tesing-Sum of Squares Error	0.282	0.461	0.223	0.252	0.467	1.162	2.606	1.331	0.474	0.184	0.277	0.182	
Testing-Relative Error	0.446	0.324	0.158	0.931	0.380	0.510	0.537	0.861	0.315	0.122	0.253	0.368	
Independent Variable Importance	%	%	%	%	%	%	%	%	%	%	%	%	
HS-100	7.9	48.0	42.5	**100.0**	49.6	31.7	60.7	21.9	15.3	40.9	37.4	17.1	
HS-200	32.4	44.8	38.3	13.7	45.4	26.7	35.8	19.9	19.6	29.3	11.3	14.2	
HS-300	24.1	50.6	68.6	67.1	**100.0**	15.6	80.6	19.0	46.9	85.9	13.9	13.1	
Albedo	29.5	23.1	96.0	14.8	96.6	42.6	53.1	27.6	19.6	47.0	13.3	29.1	
Temp	61.0	22.7	75.5	73.1	86.5	33.2	57.1	31.7	27.4	36.7	75.9	18.1	
RH	**100.0**	**100.0**	**100.0**	84.6	93.6	**100.0**	**100.0**	**100.0**	**100.0**	**100.0**	42.7	**100.0**	
*u*-Wind	17.5	8.2	61.7	18.7	42.1	27.9	91.3	54.9	34.2	23.1	30.9	17.9	
*v*-Wind	40.6	17.8	33.7	19.4	43.0	17.5	64.7	35.0	19.0	20.4	**100.0**	24.4	
TPW	7.7	38.9	20.5	38.3	35.4	30.1	16.9	3.6	8.6	32.8	16.5	8.0	

Numerous factors contributed to these findings. Previous studies have considered rainfall and RH as the crucial meteorological parameters that affect atmospheric trace element concentrations, particularly for Cu, Pb, Ni, Cr, Cd, As, and Hg [113, 114]. Anthropogenic activities in Australia and South America are also responsible for the contamination of some elements, such as Cd, Sb, Cu, As, and Pb in a 4-m snow pit at Dome Argus (Dome A) on the East Antarctic Plateau, for the period from 1950 to 2016 through atmospheric deposition [115]. Wet deposition has been recognised as one of the long-term depletion processes of elements in the ambient air of Germany [116]. Comparison of the dry and wet deposition of particulate matter in near-surface waters during summer also underlines the effectiveness of wet deposition for PM_2.5_ removal with 92% efficiency [117]. In contrast, dry deposition (63%) was more effective at removing PM_10_ than wet deposition (37%) [117]. Second, several studies have indicated forest fires, agricultural waste burning, and other open burning activities as major sources of particulate elements [118]. Sugarcane burning is considered a main contributor of Zn and Cu in aerosols because these elements are essential in superior plants and can be released from the leaves into the atmosphere [118, 119]. The presence of several soil elements, such as Na, K, Mg, and S, in bushfire-originated smoke particles emitted during different prescribed burning events in the Toohey forest region, Queensland, Australia, was also consistent with the comparatively high percentage contributions of the independent variable importance of these elements in this study [120]. Thus, we conclude that RH and hotspot number are two meteorological parameters governing the PM_2.5_-bound elements in the ambient air of Thailand.

Further attempts have been undertaken by carefully investigating the percentage contributions of independent variable importance of each selected element using ANN as displayed in Table 4. TPW is the least affecting weather variable controlling the alterations of atmospheric concentrations of S, Cl, K, V, Mn, Zn, As, Se, Br and Pb. Although the precipitation (i.e. rain and snow) is widely considered as a crucial mechanism for increasing the washout rates of aerosols, recent studies have highlighted that TPW can produce negative as well as positive impacts on PM_2.5_ contents [121]. For instance, the three precipitation episodes in both Jinan and Qingdao cities in Shandong Province, China, enhances in the source releases and diminution in the atmospheric vertical convection, which subsequently responsible in an increase in the PM_2.5_ contents [121]. An earlier study targeted on the discrepancies in the elimination impact of TPW on PM_2.5_ under various precipitation levels and pollution magnitudes [122]. The findings underline that both TPW intensity and aerosol amount influenced the washout effect. A negative washout effect presented for both low TWP and low PM_2.5_ mass content conditions. On the contrary, a positive washout effect existed for both high TWP and high PM_2.5_ mass content conditions [122].

### Risk assessment of PM_2.5_-bound elements

All HQ values detected at the three sites were <1 for both adolescents and adults (Table 5). This indicates that there is a low probability of non-cancer health risks emerging. Despite these comparatively small possibilities for non-cancer health risks, As showed the highest average HQ values detected at two air quality observatory sites for both adolescents (BOS: 0.225±0.128; POS: 0.0647±0.0551) and adults (BOS: 0.112±0.0696; POS: 0.0351±0.0299). The HQ of trace elements detected at BOS and POS decreased in the order As>Ni>Pb>Cu>Zn>Co. In contrast, the trend for the mean concentrations of trace elements measured at COS was Ni>As>Cu>Pb>Zn>Co. Co exhibited the least average values of HQ at all sampling sites. These sequences were consistent with those of previous studies conducted at Pusat Pembangunan Tenaga Industri Johor (PUSPATRI) in Pasir Gudang, Universiti Tun Hussein Onn Malaysia (UTHM) in Batu Pahat, and Sekolah Menengah Kebangsaan Gelang Patan (SMKGP) in Gelang Patah, Malaysia [52]. The decreasing order of the carcinogenic elements detected at PUSPATRI, UTHM, and SMKGP by their average HQ values were As>Ni>Pb>Cu>Zn>Co for both age groups [52]. A similar study conducted in urban areas of Tehran, Iran, in the middle of the summer period exhibited the decreasing order of HQ as As>Pb>Cu>Ni>Zn [123]. This result was surprisingly consistent with this study and a previous one [52], where As and Zn had the largest and smallest HQ values, respectively. As showed the highest average HQ value in all the sampling sites (i.e. COS, BOS, and POS) for both adolescents and adults, with the value of the adolescent (average = 0.123) to be 1.8 times greater than that of the adults (average = 0.0667). Average HQ values of As measured at COS, BOS, and POS were compared for further investigations. The average HQ value of As detected at BOS (0.225) was 2.6 times and 3.5 times higher than at COS (0.0788) and POS (0.0647), respectively. Even though all HQ values of As and other carcinogenic elements fell in the range of under risk (<1), the adolescents in this study, i.e. college students of Suan Sunandha Rajabhat University (i.e. BOS), should theoretically pay more attention to their adverse health impacts. According to the Enhancement and Conservation of the National Environmental Quality Act 1992 (B.E.2535), which is a central law on the protection of the environment in Thailand, Pb is the only element registered in the National Ambient Air Quality Standards (NAAQSs) in Thailand with a 1-month average of 0.0015 mg m^-3^ [124]. Hence, policymakers should consider whether to include As in the elements registered in NAAQSs due to its comparatively high HQ values, particularly in the ambient air of Bangkok.

**Table 5:** Statistical description of hazard quotient (HQ) for adolescents and adults exposed to some heavy metals in PM_2.5_ collected at COS, BOS, and POS.

		Adolescent						Adult					
	Site	Co	Ni	Cu	Zn	As	Pb	Co	Ni	Cu	Zn	As	Pb
Aver	COS	9.15E-05	1.70E-01	7.63E-04	2.99E-04	7.88E-02	7.45E-03	4.96E-05	9.22E-02	4.14E-04	1.62E-04	4.28E-02	4.05E-03
Stdev	(*n* = 82)	7.18E-06	1.70E-02	9.42E-04	1.50E-04	4.07E-02	3.23E-03	3.90E-06	9.20E-03	5.11E-04	8.14E-05	2.21E-02	1.76E-03
Min		7.63E-05	1.40E-01	3.18E-04	1.53E-04	N.D.	3.27E-03	4.14E-05	7.60E-02	1.73E-04	8.29E-05	N.D.	1.78E-03
Max		1.14E-04	2.29E-01	6.33E-03	8.78E-04	1.78E-01	2.04E-02	6.22E-05	1.24E-01	3.44E-03	4.77E-04	9.67E-02	1.11E-02
Aver	BOS	1.34E-04	1.98E-01	1.54E-03	1.18E-03	2.25E-01	1.87E-02	7.25E-05	1.08E-01	8.37E-04	6.42E-04	1.22E-01	1.01E-02
Stdev	(*n* = 48)	1.29E-05	9.12E-02	1.32E-03	4.83E-04	1.28E-01	1.06E-02	7.01E-06	4.95E-02	7.17E-04	2.62E-04	6.96E-02	5.76E-03
Min		8.59E-05	9.54E-02	8.11E-04	4.39E-04	N.D.	7.09E-03	4.66E-05	5.18E-02	4.40E-04	2.38E-04	N.D.	3.85E-03
Max		1.53E-04	3.82E-01	9.16E-03	2.56E-03	6.87E-01	6.00E-02	8.29E-05	2.07E-01	4.97E-03	1.39E-03	3.73E-01	3.26E-02
Aver	POS	9.28E-05	5.87E-02	6.77E-04	2.51E-04	6.47E-02	4.74E-03	5.04E-05	3.19E-02	3.68E-04	1.36E-04	3.51E-02	2.57E-03
Stdev	(*n* = 61)	6.91E-06	1.13E-02	4.49E-04	7.75E-05	5.51E-02	1.31E-03	3.75E-06	6.13E-03	2.44E-04	4.21E-05	2.99E-02	7.12E-04
Min		7.63E-05	4.45E-02	3.50E-04	1.82E-04	N.D.	2.54E-03	4.14E-05	2.42E-02	1.90E-04	9.90E-05	N.D.	1.38E-03
Max		1.08E-04	1.21E-01	3.78E-03	6.40E-04	3.56E-01	1.24E-02	5.87E-05	6.56E-02	2.05E-03	3.48E-04	1.93E-01	6.71E-03

In this study, the ELCR values of adolescents and adults living adjacent to COS, BOS, and POS were estimated using IUR, SF, and LADD (Eqs 3–4). Unlike the HQ values, the adult age group exhibited higher ELCR values for all elements (i.e. As, Ni, and Pb) than the adolescent age group, which is consistent with earlier reports [52, 123]. ELCR values decreased in the order of As>Ni>Pb at all three sites for both the adolescent and adult age groups (Table 6). This sequence is consistent with a previous study conducted at PUSPATRI, UTHM, and SMKGP in Malaysia [52]. Even though the ELCR values of all elements fall within an acceptable range, the adult age group was more vulnerable to cancer-associated adverse health impacts compared to the adolescent age group (Table 6). Impacts of geographical conditions on the average ELCR values determined at COS, BOS, and POS were further assessed. One-way ANOVA was employed to determine any statistically significant differences between the mean ELCR values detected at the three sites. The ELCR values of Ni (*F* = 142; *F*_crit_ = 3.04; *p* <0.05), As (*F* = 72.5; *F*_crit_ = 3.04; *p* <0.05), and Pb (*F* = 86.7; *F*_crit_ = 3.04; *p* <0.05) observed at the BOS were significantly higher than those at the other two sites. Since previous studies underline traffic emissions as the predominant potential source of particulate elements in ambient air in Bangkok [12, 14], numerous incentives for air pollution control measures, such as favourable tax treatment for catalytic converters and diesel particulate filters attached to mufflers of both light and heavy-duty vehicles, are highly recommended to eliminate harmful particulate elements from vehicle exhausts.

**Table 6:** Statistical description of excess lifetime cancer risk (ELCR) for adolescents and adults exposed to some heavy metals in PM_2.5_ collected at COS, BOS, and POS.

		Adolescent			Adult		
	Site	Ni	As	Pb	Ni	As	Pb
Aver	COS	1.69E-06	3.51E-06	6.48E-08	6.76E-06	1.41E-05	2.60E-07
Stdev	(*n* = 82)	1.69E-07	1.81E-06	2.81E-08	6.75E-07	7.27E-06	1.13E-07
Min		1.39E-06	N.D.	2.85E-08	5.57E-06	N.D.	1.14E-07
Max		2.28E-06	7.94E-06	1.77E-07	9.12E-06	3.18E-05	7.09E-07
Aver	BOS	1.97E-06	1.00E-05	1.62E-07	7.89E-06	4.02E-05	6.50E-07
Stdev	(*n* = 48)	9.09E-07	5.72E-06	9.23E-08	3.63E-06	2.29E-05	3.70E-07
Min		9.50E-07	N.D.	6.17E-08	3.80E-06	N.D.	2.47E-07
Max		3.80E-06	3.06E-05	5.22E-07	1.52E-05	1.23E-04	2.09E-06
Aver	POS	5.85E-07	2.88E-06	4.12E-08	2.34E-06	1.16E-05	1.65E-07
Stdev	(*n* = 61)	1.12E-07	2.45E-06	1.14E-08	4.49E-07	9.84E-06	4.57E-08
Min		4.43E-07	N.D.	2.21E-08	1.77E-06	N.D.	8.87E-08
Max		1.20E-06	1.59E-05	1.08E-07	4.81E-06	6.36E-05	4.31E-07

## Conclusions

PCA indicated traffic emissions as the greatest contributors of particulate elements (i.e. Pb, Zn, Br, As, and Ni) in all the monitoring sites. These findings are consistent with those of earlier studies conducted at the same sites and with the employment of *EFs*. Although strong positive correlations of Na, Mg, and Si imply significant crustal inputs coupled with maritime aerosols, some high correlation coefficients of Ca, Sc, Ti, Mn, Fe, and Sr suggest the influence of construction dust on the variations in PM_2.5_ contents. Application of the diagnostic ratios of some elements (i.e. Ca/Al and Ca/Si) also highlights the regular impact of construction dust on the air quality level of the three monitoring sites. Some significant seasonal differences in Ni/V, V/Fe, and As/V indicate the inevitable repercussions of open burning on air quality deterioration, particularly in the middle of a haze episode. ANN models suggest that relative humidity and hotspot numbers are the two main factors controlling the concentrations of elements in PM_2.5_ in the ambient air of Chiang-Mai, Bangkok, and Phuket. Although the ELCR values of all elements fell within an acceptable range, the adult age group was associated with a higher risk of developing cancer than the adolescent group. To sustainably reduce the adverse health impacts of air pollutants, a comprehensive air quality management policy and program is essential. Several economic incentives, such as tax cuts and subsidies to promote electric vehicle, tax rebates for investing in solar energy, non-agricultural burning subsidy, and negative economic incentives for punishing polluters, should be included in the environmental quality management plan under Section 35 of the promotion and conservation of national environmental quality act (‘NEQA’) B.E. 2535.

## Supporting information

S1 Fig Hot spot number detected in Thailand, Vietnam, Myanmar, Cambodia, and Lao PDR from 2020 to 2021 reported by the Geo-Informatics and Space Technology Development Agency (GISTDA). (TIF)

S1 Table Diagnostic binary ratios of predicted source contributions (P) to measured concentrations (M) of 25 selected metals for each sample. (PDF)

S2 Table Exposure factors for computing ADD and LADD in health risk assessment related with heavy metals in PM_2.5_ collected at COS, BOS, and POS. (PDF)

S3 Table Reference dose (RfD), inhalation unit risk (IUR) and slope factor (SF) for computing HQ and ELCR in health risk assessment associated with heavy metals in PM_2.5_ collected at COS, BOS, and POS. (PDF)

S4 Table ADD for computing HQ in health risk assessment associated with heavy metals in PM_2.5_ collected at COS, BOS, and POS. (PDF)

S5 Table Pearson correlation coefficients of heavy metals in PM_2.5_ collected at COS. (PDF)

S6 Table Pearson correlation coefficients of heavy metals in PM_2.5_ collected at BOS. (PDF)

S7 Table Pearson correlation coefficients of heavy metals in PM_2.5_ collected at POS. (PDF)
