# Correction: Combined use of principal component analysis/multiple linear regression analysis and artificial neural network to assess the impact of meteorological parameters on fluctuation of selected PM_2.5_-bound elements

**DOI:** 10.1371/journal.pone.0302975

**Published:** 2024-04-30

**Authors:** Siwatt Pongpiachan, Qiyuan Wang, Ronbanchob Apiratikul, Danai Tipmanee, Li Li, Li Xing, Xingli Mao, Guohui Li, Yongming Han, Junji Cao, Vanisa Surapipith, Aekkapol Aekakkararungroj, Saran Poshyachinda

An additional affiliation is missing for the first author. Siwatt Pongpiachan is also affiliated with the National Astronomical Research Institute of Thailand (Public Organization), Chiangmai, Thailand.

The correct affiliations are as follows:

Siwatt Pongpiachan^1,2^, Qiyuan Wang^3^, Ronbanchob Apiratikul^4^, Danai Tipmanee^5^, Li Li^3^, Li Xing^6^, Xingli Mao^6^, Guohui Li^3^, Yongming Han^3^, Junji Cao^3^, Vanisa Surapipith^1^, Aekkapol Aekakkararungroj^7^, Saran Poshyachinda^1^

**1** National Astronomical Research Institute of Thailand (Public Organization), Chiangmai, Thailand, **2** NIDA Center for Research & Development of Disaster Prevention & Management, School of Social and Environmental Development, National Institute of Development Administration (NIDA), Bangkok, Thailand, **3** State Key Laboratory of Loess and Quaternary Geology, Institute of Earth Environment, Chinese Academy of Sciences (IEECAS), Xi’an, China, **4** Faculty of Science, Suansunandha Rajabhat, Bangkok, Thailand, **5** Faculty of Technology and Environment, Prince of Songkla University, Phuket, Thailand, **6** School of Geography and Tourism, Shaanxi Normal University, Xi’an, China, **7** Asian Disaster Preparedness Center (ADPC), Bangkok, Thailand.
