# Supplementary figures and images for: Combined use of principal component analysis/multiple linear regression analysis and artificial neural network to assess the impact of meteorological parameters on fluctuation of selected PM2.5-bound elements
Source: PLoS One. 2024 Mar 20;19(3):e0287187. doi: 10.1371/journal.pone.0287187 (PMC10954151; doi:10.1371/journal.pone.0287187)

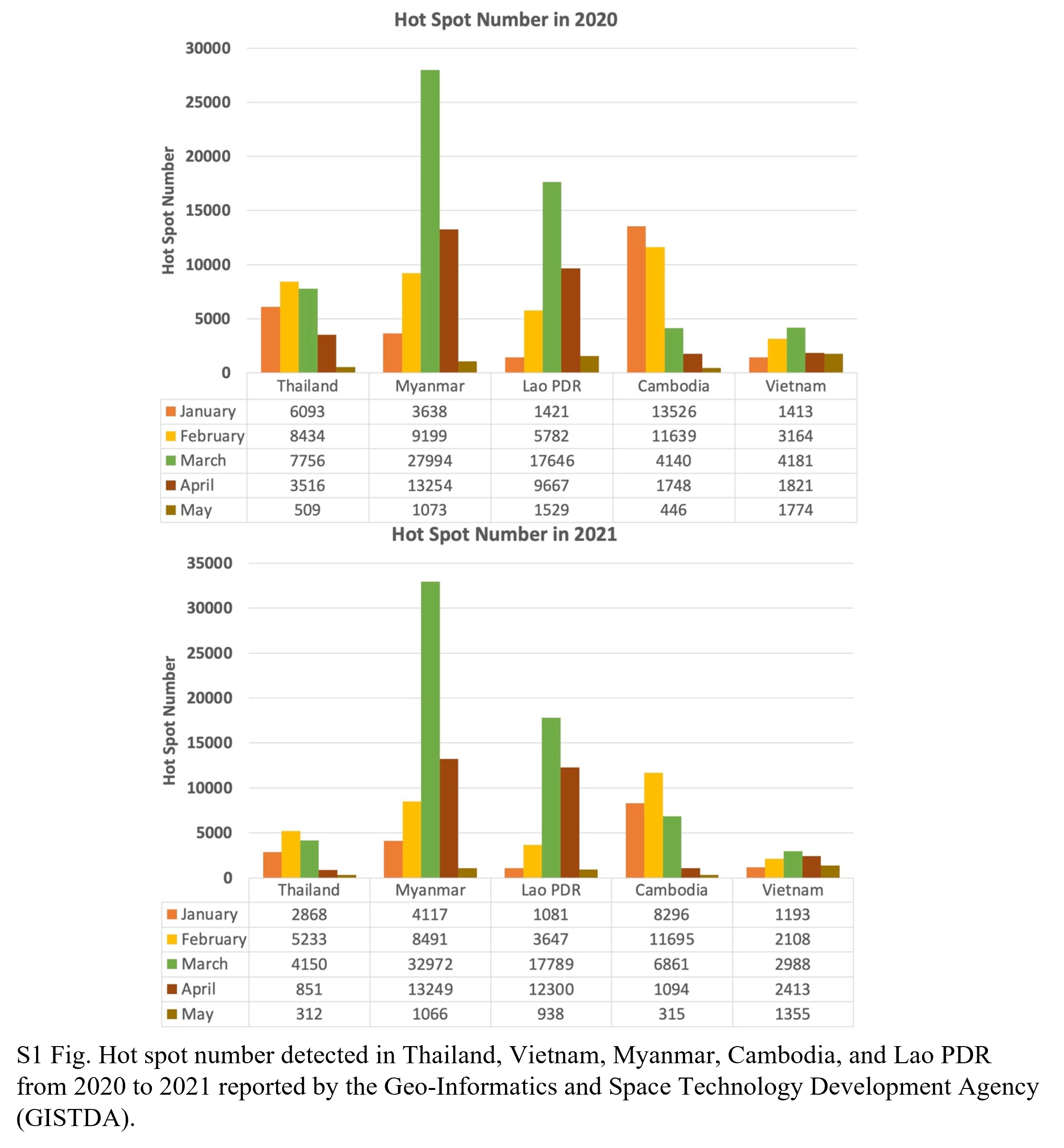

Supplement: S1 Fig — (TIF) [file pone.0287187.s001.tif]
