# Supplementary material for: Combined use of principal component analysis/multiple linear regression analysis and artificial neural network to assess the impact of meteorological parameters on fluctuation of selected PM2.5-bound elements
Source: PLoS One. 2024 Mar 20;19(3):e0287187. doi: 10.1371/journal.pone.0287187 (PMC10954151; doi:10.1371/journal.pone.0287187)
Supplement: S1 Table — (PDF) [file pone.0287187.s002.pdf]

S1 Table. Diagnostic binary ratios of predicted source contributions (P) to measured concentrations (M) of 25 selected metals for each sample

Sample	COS			BOS				POS			
Date	M[a]	P[b]	P/M[c]	Date	M	P	P/M	Date	M	P	P/M
3/7/2020	1894	1981	1.05	5/12/2020	4577	4460	0.97	1/8/2020	1895	1948	1.03
7/7/2020	1799	1930	1.07	7/12/2020	5035	5092	1.01	3/8/2020	1839	1938	1.05
11/7/2020	1938	1919	0.99	14/12/2020	5742	5567	0.97	7/8/2020	2373	2421	1.02
15/7/2020	1846	1971	1.07	19/12/2020	3634	3336	0.92	16/9/2020	1936	1887	0.97
19/7/2020	1814	1971	1.09	22/12/2020	3693	3816	1.03	20/9/2020	2053	1950	0.95
23/7/2020	1963	1938	0.99	26/12/2020	3560	3624	1.02	25/9/2020	1982	1942	0.98
27/7/2020	1924	1930	1.00	29/12/2020	4617	4743	1.03	28/9/2020	1978	1895	0.96
31/7/2020	2166	2084	0.96	2/1/2021	3798	3512	0.92	2/10/2020	1904	1878	0.99
4/8/2020	1936	1980	1.02	5/1/2021	4459	4323	0.97	7/10/2020	2137	1998	0.93
8/8/2020	2003	1965	0.98	9/1/2021	3514	3490	0.99	12/10/2020	1947	1967	1.01
12/8/2020	1833	1969	1.07	12/1/2021	4381	4317	0.99	17/10/2020	1902	1928	1.01
16/8/2020	2578	2795	1.08	16/1/2021	4126	4036	0.98	26/10/2020	1807	1910	1.06
20/8/2020	1833	1938	1.06	19/1/2021	5195	5103	0.98	30/10/2020	1927	1896	0.98
24/8/2020	1850	1940	1.05	23/1/2021	4082	4093	1.00	6/11/2020	2004	1989	0.99
28/8/2020	1971	1919	0.97	26/1/2021	3805	3806	1.00	11/11/2020	2140	2139	1.00
1/9/2020	2021	1989	0.98	30/1/2021	4021	3940	0.98	23/11/2020	2069	2094	1.01
5/9/2020	1927	1928	1.00	2/2/2021	4900	4946	1.01	30/11/2020	1898	1891	1.00
9/9/2020	1990	1946	0.98	6/2/2021	4441	4484	1.01	5/12/2020	1814	1930	1.06
13/9/2020	1894	1903	1.00	9/2/2021	4104	4063	0.99	16/12/2020	1788	1886	1.06
17/9/2020	1938	2066	1.07	13/2/2021	3109	3182	1.02	21/12/2020	2517	2429	0.97
21/9/2020	1783	1933	1.08	16/2/2021	3695	3605	0.98	25/12/2020	1835	1880	1.02
25/9/2020	2174	2275	1.05	20/2/2021	4204	4223	1.00	4/1/2021	1887	1896	1.00
29/9/2020	1973	1906	0.97	23/2/2021	3546	3711	1.05	11/1/2021	1835	1869	1.02
3/9/2020	1903	1928	1.01	27/2/2021	5362	4927	0.92	15/1/2021	2065	1917	0.93
7/10/2020	2013	1957	0.97	2/3/2021	3154	3277	1.04	19/1/2021	2014	1933	0.96
11/10/2020	1956	2064	1.06	6/3/2021	3680	3423	0.93	26/1/2021	1932	1876	0.97
15/10/2020	2017	1920	0.95	13/3/2021	3964	3726	0.94	5/2/2021	1917	1891	0.99
19/10/2020	2080	2016	0.97	23/3/2021	3421	3479	1.02	11/2/2021	1879	1880	1.00
23/10/2020	2098	2018	0.96	3/4/2021	3744	3720	0.99	28/2/2021	1988	1939	0.98
27/10/2020	2333	2279	0.98	30/3/2021	3072	3203	1.04	15/2/2021	2008	1929	0.96
31/10/2020	1944	1952	1.00	27/3/2021	3669	4342	1.18	23/2/2021	1793	1905	1.06
4/11/2020	1860	1925	1.03	9/3/2021	3240	3177	0.98	2/3/2021	2064	2184	1.06
8/11/2020	2087	2011	0.96	16/3/2021	3780	3612	0.96	6/3/2021	1959	2057	1.05
12/11/2020	2082	2060	0.99	20/3/2021	3687	3788	1.03	10/3/2021	1875	1911	1.02
16/11/2020	1972	2038	1.03	24/4/2021	2977	3111	1.04	14/3/2021	1854	1871	1.01
20/11/2020	2398	2391	1.00	15/5/2021	4536	4979	1.10	18/3/2021	1912	1911	1.00
24/11/2020	2155	2011	0.93	11/5/2021	3085	3136	1.02	22/3/2021	1805	1910	1.06
28/11/2020	2396	2363	0.99	19/6/2021	4168	4169	1.00	26/3/2021	2042	1891	0.93
2/12/2020	2375	2263	0.95	15/6/2021	3725	3913	1.05	30/3/2021	1922	1872	0.97
7/12/2020	2149	2070	0.96	1/6/2021	3564	3668	1.03	3/4/2021	1970	1967	1.00
11/12/2020	2035	2000	0.98	8/5/2021	4008	3824	0.95	7/4/2021	1867	1913	1.02
15/12/2020	1902	1976	1.04	29/5/2021	4026	4374	1.09	11/4/2021	1860	1906	1.02
19/12/2020	1927	1972	1.02	5/5/2021	3424	3687	1.08	15/4/2021	1878	1896	1.01
23/12/2020	1892	1916	1.01	26/4/2021	3648	3714	1.02	19/4/2021	1838	1881	1.02
27/12/2020	2401	2290	0.95	28/4/2021	4082	3989	0.98	23/4/2021	1791	1868	1.04
31/12/2020	1988	1987	1.00	1/5/2021	3622	3499	0.97	27/4/2021	1876	1864	0.99
4/1/2021	2011	1947	0.97	20/4/2021	4992	4753	0.95	1/5/2021	1898	1900	1.00
8/1/2021	2073	2000	0.96	7/4/2021	4546	4448	0.98	5/5/2021	1859	1885	1.01
12/1/2021	2091	2084	1.00					9/5/2021	1947	1928	0.99
16/1/2021	2169	2271	1.05					13/5/2021	1968	1976	1.00

20/1/2021	2247	2309	1.03	17/5/2021	1965	1973	1.00
24/1/2021	2174	2283	1.05	21/5/2021	1990	1945	0.98
28/1/2021	2213	2167	0.98	25/5/2021	1884	1867	0.99
1/2/2021	2187	2047	0.94	29/5/2021	1904	1936	1.02
5/2/2021	2227	2144	0.96	2/6/2021	1905	1866	0.98
9/2/2021	2035	2014	0.99	6/6/2021	1907	1919	1.01
13/2/2021	2202	2043	0.93	10/6/2021	1885	1943	1.03
17/2/2021	2316	2091	0.90	14/6/2021	1937	1916	0.99
23/2/2021	2597	2508	0.97	18/6/2021	2002	1945	0.97
27/2/2021	2637	2556	0.97	22/6/2021	2138	2005	0.94
3/3/2021	3001	3006	1.00	26/6/2021	1808	1872	1.04
7/3/2021	2694	2792	1.04				
11/3/2021	2240	2401	1.07				
15/3/2021	2398	2431	1.01				
19/3/2021	2611	2463	0.94				
23/3/2021	3136	3075	0.98				
28/3/2021	2740	2766	1.01				
1/4/2021	2867	2627	0.92				
5/4/2021	2054	1977	0.96				
9/4/2021	2001	2023	1.01				
13/4/2021	1957	2104	1.08				
17/4/2021	1967	1985	1.01				
21/4/2021	2154	2250	1.04				
25/4/2021	2060	2159	1.05				
29/4/2021	2062	1926	0.93				
4/5/2021	2003	1936	0.97				
8/5/2021	2010	1962	0.98				
12/5/2021	1992	2072	1.04				
16/5/2021	2065	2129	1.03				
20/5/2021	1946	1981	1.02				
24/5/2021	2049	1944	0.95				
28/5/2021	1803	1917	1.06				

[a]Measured concentration: sum of measured 25 metal concentrations in each sample.
[b]Predicted: sum of contributions of three principal components (estimated the sum of 25 metal concentrations)
[c]Predicted/Measured: the binary ratio of estimated the sum of 25 metal concentrations to the measured sum of 25 metal concentrations
