# Supplementary material for: Combined use of principal component analysis/multiple linear regression analysis and artificial neural network to assess the impact of meteorological parameters on fluctuation of selected PM2.5-bound elements
Source: PLoS One. 2024 Mar 20;19(3):e0287187. doi: 10.1371/journal.pone.0287187 (PMC10954151; doi:10.1371/journal.pone.0287187)
Supplement: S2 Table — (PDF) [file pone.0287187.s003.pdf]

S2 Table. Exposure factors for computing ADD and LADD in health risk assessment related with heavy metals in $PM_{2.5}$ collected at COS, BOS, and POS

**Exposure factors**	**Unit**	**Adolescent**	**Adult**	**Reference**
IR (Inhalation rate)	m^3 day^{-1}	12<18 years	18<70 years	EPA (2001)
ED (Exposure dose)	Years	6	24	EPA (2001)
EF (Exposure frequency)	day $year^{-1}$	350	350	EPA (2001)
BW (Body weight)	Kg	38	70	EPA (2001)
AT (Averaging time) for non-carcinogens	Days	2,190	8,760	EPA (2001)
AT (Averaging time) for carcinogens	Days	25,550	25,550	EPA (2001)
