# Supplementary material for: Combined use of principal component analysis/multiple linear regression analysis and artificial neural network to assess the impact of meteorological parameters on fluctuation of selected PM2.5-bound elements
Source: PLoS One. 2024 Mar 20;19(3):e0287187. doi: 10.1371/journal.pone.0287187 (PMC10954151; doi:10.1371/journal.pone.0287187)
Supplement: S3 Table — (PDF) [file pone.0287187.s004.pdf]

S3 Table. Reference dose (RfD), inhalation unit risk (IUR) and slope factor (SF) for computing HQ and ELCR in health risk assessment associated with heavy metals in $PM_{2.5}$ collected at COS, BOS, and POS

**Selected metals**	**RfD (ng kg^{-1} day^{-1})**	**IUR (m^3 μg^{-1})**	**SF (day kg μg^{-1})**	**SF (day kg μg^{-1})**
			Adolescent	Adult
Co	200,000			
Ni	200	0.00024	0.000581	0.00107
Cu	40,000			
Zn	300,000			
As	50	0.0043	0.0104	0.0192
Pb	3,500	0.000012	0.000029	0.0000535

Source: EPA (2005); Granero and Domingo (2002); Peña-Fernández et al. (2014)
