# Supplementary material for: Combined use of principal component analysis/multiple linear regression analysis and artificial neural network to assess the impact of meteorological parameters on fluctuation of selected PM2.5-bound elements
Source: PLoS One. 2024 Mar 20;19(3):e0287187. doi: 10.1371/journal.pone.0287187 (PMC10954151; doi:10.1371/journal.pone.0287187)
Supplement: S4 Table — (PDF) [file pone.0287187.s005.pdf]

S4 Table. ADD for computing HQ in health risk assessment associated with heavy metals in $PM_{2.5}$ collected at COS, BOS, and POS

		Adolescent								Adult							
	Site	V	Co	Ni	Cu	Zn	As	Se	Pb	V	Co	Ni	Cu	Zn	As	Se	Pb
Aver	POS	3.88E+00	1.86E+01	1.17E+01	2.71E+01	7.54E+01	3.23E+00	1.44E+00	1.66E+01	2.11E+00	1.01E+01	6.37E+00	1.47E+01	4.09E+01	1.75E+00	7.81E-01	9.00E+00
Stdev		9.70E-01	1.38E+00	2.26E+00	1.79E+01	2.32E+01	2.75E+00	5.91E-01	4.59E+00	5.26E-01	7.50E-01	1.23E+00	9.74E+00	1.26E+01	1.49E+00	3.21E-01	2.49E+00
Min		1.27E+00	1.53E+01	8.91E+00	1.40E+01	5.47E+01	0.00E+00	1.27E+00	8.91E+00	6.91E-01	8.29E+00	4.83E+00	7.60E+00	2.97E+01	0.00E+00	6.91E-01	4.83E+00
Max		6.36E+00	2.16E+01	2.42E+01	1.51E+02	1.92E+02	1.78E+01	5.09E+00	4.33E+01	3.45E+00	1.17E+01	1.31E+01	8.22E+01	1.04E+02	9.67E+00	2.76E+00	2.35E+01
Aver	COS	3.80E+00	1.83E+01	3.40E+01	3.05E+01	8.96E+01	3.94E+00	1.52E+00	2.61E+01	2.06E+00	9.93E+00	1.84E+01	1.66E+01	4.87E+01	2.14E+00	8.25E-01	1.42E+01
Stdev		1.05E+00	1.44E+00	3.39E+00	3.77E+01	4.50E+01	2.03E+00	5.07E-01	1.13E+01	5.69E-01	7.80E-01	1.84E+00	2.05E+01	2.44E+01	1.10E+00	2.75E-01	6.14E+00
Min		1.27E+00	1.53E+01	2.80E+01	1.27E+01	4.58E+01	0.00E+00	1.27E+00	1.14E+01	6.91E-01	8.29E+00	1.52E+01	6.91E+00	2.49E+01	0.00E+00	6.91E-01	6.22E+00
Max		6.36E+00	2.29E+01	4.58E+01	2.53E+02	2.63E+02	8.91E+00	2.54E+00	7.12E+01	3.45E+00	1.24E+01	2.49E+01	1.37E+02	1.43E+02	4.83E+00	1.38E+00	3.87E+01
Aver	BOS	7.16E+00	2.67E+01	3.96E+01	6.17E+01	3.55E+02	1.13E+01	6.44E+00	6.53E+01	3.88E+00	1.45E+01	2.15E+01	3.35E+01	1.93E+02	6.11E+00	3.50E+00	3.55E+01
Stdev		2.35E+00	2.58E+00	1.82E+01	5.28E+01	1.45E+02	6.41E+00	4.65E+00	3.71E+01	1.27E+00	1.40E+00	9.91E+00	2.87E+01	7.87E+01	3.48E+00	2.53E+00	2.02E+01
Min		1.91E+00	1.72E+01	1.91E+01	3.24E+01	1.32E+02	0.00E+00	1.91E+00	2.48E+01	1.04E+00	9.32E+00	1.04E+01	1.76E+01	7.15E+01	0.00E+00	1.04E+00	1.35E+01
Max		1.34E+01	3.05E+01	7.63E+01	3.66E+02	7.69E+02	3.43E+01	2.48E+01	2.10E+02	7.25E+00	1.66E+01	4.14E+01	1.99E+02	4.17E+02	1.86E+01	1.35E+01	1.14E+02
