# Supplementary material for: Combined use of principal component analysis/multiple linear regression analysis and artificial neural network to assess the impact of meteorological parameters on fluctuation of selected PM2.5-bound elements
Source: PLoS One. 2024 Mar 20;19(3):e0287187. doi: 10.1371/journal.pone.0287187 (PMC10954151; doi:10.1371/journal.pone.0287187)
Supplement: S5 Table — (PDF) [file pone.0287187.s006.pdf]

S5 Table. Pearson correlation coefficients of heavy metals in $PM_{2.5}$ collected at COS

	Na	Mg	Al	Si	S	Cl	K	Ca	Sc	Ti	V	Cr	Mn	Fe	Co	Ni	Cu	Zn	Ga	As	Se	Br	Sr	Ba
Na	1.000																							
Mg	**0.984**	1.000																						
Al	0.479	0.559	1.000																					
Si	**0.922**	**0.901**	0.309	1.000																				
S	0.164	0.125	0.063	-0.169	1.000																			
Cl	-0.185	-0.171	-0.092	-0.267	0.188	1.000																		
K	0.084	0.051	0.084	-0.218	**0.880**	0.405	1.000																	
Ca	0.017	0.043	-0.083	-0.110	0.288	0.394	0.289	1.000																
Sc	0.079	0.141	0.102	0.099	-0.152	0.038	-0.126	0.259	1.000															
Ti	0.121	0.118	0.032	-0.180	**0.869**	0.286	0.796	0.410	0.055	1.000														
V	-0.081	-0.086	-0.116	-0.082	0.079	-0.162	-0.121	0.003	-0.051	0.111	1.000													
Cr	-0.094	-0.061	0.117	-0.040	-0.125	-0.089	-0.118	-0.129	0.033	-0.159	-0.013	1.000												
Mn	0.125	0.106	0.091	-0.144	**0.823**	0.218	0.745	0.198	-0.130	**0.778**	0.085	-0.100	1.000											
Fe	0.093	0.091	0.056	-0.209	**0.888**	0.295	**0.828**	0.361	0.015	**0.976**	0.082	-0.046	**0.827**	1.000										
Co	0.209	0.245	0.157	0.244	-0.136	-0.100	-0.133	0.094	0.249	-0.045	-0.010	0.037	-0.184	-0.083	1.000									
Ni	-0.037	-0.040	-0.230	0.102	-0.178	0.255	-0.082	0.083	0.162	-0.111	-0.154	0.064	-0.064	-0.081	0.059	1.000								
Cu	0.000	-0.013	-0.006	-0.036	0.173	0.266	0.313	0.011	-0.060	0.115	-0.160	-0.035	0.154	0.146	-0.078	0.210	1.000							
Zn	0.075	0.074	0.072	-0.170	**0.730**	0.335	0.702	0.336	0.052	**0.780**	-0.004	-0.127	**0.752**	**0.812**	-0.172	0.187	0.160	1.000						
Ga	0.197	0.191	0.050	0.150	0.212	-0.176	0.145	0.048	0.183	0.200	0.133	-0.079	0.158	0.201	0.019	-0.003	-0.182	0.153	1.000					
As	-0.078	-0.096	-0.036	-0.215	0.460	0.408	0.508	0.068	-0.084	0.474	0.029	-0.096	0.457	0.492	-0.005	-0.019	0.116	0.400	0.168	1.000				
Se	-0.031	-0.032	0.119	-0.227	0.498	0.379	0.601	0.163	0.089	0.526	-0.180	-0.109	0.439	0.537	0.083	-0.164	0.035	0.462	0.036	0.473	1.000			
Br	0.060	0.038	0.072	-0.240	**0.849**	0.358	**0.941**	0.259	-0.100	**0.829**	-0.122	-0.152	**0.730**	**0.850**	-0.109	-0.198	0.211	**0.704**	0.130	0.500	0.641	1.000		
Sr	0.127	0.158	0.018	-0.058	0.514	0.328	0.505	0.530	0.334	0.670	0.115	-0.177	0.502	0.653	0.123	0.083	0.099	0.530	0.259	0.264	0.381	0.530	1.000	
Ba	-0.193	-0.187	-0.119	-0.013	-0.400	-0.019	-0.324	-0.314	-0.113	-0.461	-0.037	0.078	-0.302	-0.399	-0.142	0.280	-0.019	-0.261	0.073	-0.049	-0.243	-0.352	-0.151	1.000

*Please note that any value higher than 0.7 was highlighted in bold font
