# Supplementary material for: Combined use of principal component analysis/multiple linear regression analysis and artificial neural network to assess the impact of meteorological parameters on fluctuation of selected PM2.5-bound elements
Source: PLoS One. 2024 Mar 20;19(3):e0287187. doi: 10.1371/journal.pone.0287187 (PMC10954151; doi:10.1371/journal.pone.0287187)
Supplement: S6 Table — (PDF) [file pone.0287187.s007.pdf]

S6 Table. Pearson correlation coefficients of heavy metals in $PM_{2.5}$ collected at BOS

	Na	Mg	Al	Si	S	Cl	K	Ca	Sc	Ti	V	Cr	Mn	Fe	Co	Ni	Cu	Zn	Ga	As	Se	Br	Sr	Ba
Na	1.000																							
Mg	**0.979**	1.000																						
Al	0.239	0.362	1.000																					
Si	**0.929**	**0.919**	0.202	1.000																				
S	0.255	0.178	-0.181	-0.041	1.000																			
Cl	-0.232	-0.298	-0.251	-0.340	-0.029	1.000																		
K	0.034	0.009	-0.103	-0.242	**0.739**	0.144	1.000																	
Ca	0.352	0.324	-0.089	0.192	0.274	0.303	0.104	1.000																
Sc	0.290	0.300	0.008	0.393	-0.329	0.049	-0.485	0.538	1.000															
Ti	-0.004	-0.079	-0.291	-0.106	0.149	0.434	-0.080	**0.805**	0.452	1.000														
V	-0.144	-0.142	-0.124	-0.145	-0.001	-0.099	-0.169	0.016	0.022	0.112	1.000													
Cr	-0.188	-0.164	0.193	-0.310	0.293	0.052	0.441	0.075	-0.097	0.024	-0.082	1.000												
Mn	0.129	0.074	-0.113	-0.161	**0.713**	0.224	0.631	0.554	-0.005	0.531	0.121	0.426	1.000											
Fe	0.067	-0.014	-0.208	-0.126	0.394	0.436	0.168	**0.820**	0.306	**0.932**	0.149	0.211	**0.743**	1.000										
Co	0.426	0.449	0.003	0.496	-0.050	-0.400	-0.321	0.386	0.556	0.179	0.116	-0.210	-0.097	0.062	1.000									
Ni	0.208	0.241	0.129	0.013	0.481	0.037	**0.748**	0.015	-0.387	-0.325	-0.179	0.397	0.374	-0.072	-0.232	1.000								
Cu	0.068	0.117	0.135	-0.051	0.246	0.028	0.303	0.153	-0.216	0.004	-0.134	0.225	0.199	0.069	0.003	0.290	1.000							
Zn	0.224	0.188	-0.099	0.034	0.426	0.201	0.429	0.374	0.011	0.317	0.357	0.305	**0.751**	0.511	-0.036	0.361	0.236	1.000						
Ga	-0.013	-0.069	-0.277	-0.078	0.203	0.112	0.135	0.106	-0.011	0.169	0.078	-0.058	0.337	0.218	0.100	0.156	-0.282	0.382	1.000					
As	0.108	0.082	-0.108	-0.147	**0.708**	0.104	**0.779**	0.126	-0.364	-0.056	0.035	0.429	0.586	0.181	-0.179	0.650	0.319	0.578	0.243	1.000				
Se	0.130	0.104	-0.054	0.039	0.324	-0.166	0.387	-0.063	-0.346	-0.180	-0.146	0.080	0.150	-0.084	0.018	0.404	0.438	0.142	0.171	0.418	1.000			
Br	-0.064	-0.089	-0.065	-0.340	**0.705**	0.141	**0.932**	0.025	-0.591	-0.060	-0.130	0.424	0.574	0.174	-0.402	0.617	0.306	0.344	-0.002	**0.719**	0.356	1.000		
Sr	0.153	0.136	-0.169	0.166	-0.184	0.216	-0.374	0.660	0.677	0.677	0.233	-0.094	0.111	0.520	0.497	-0.484	-0.089	0.136	0.008	-0.178	-0.325	-0.397	1.000	
Ba	-0.471	-0.486	-0.099	-0.427	-0.183	0.239	-0.226	-0.043	-0.108	0.286	0.216	0.005	-0.065	0.215	-0.258	-0.430	-0.038	-0.105	-0.041	-0.235	-0.254	-0.100	0.197	1.000

*Please note that any value higher than 0.7 was highlighted in bold font
