# Supplementary material for: Combined use of principal component analysis/multiple linear regression analysis and artificial neural network to assess the impact of meteorological parameters on fluctuation of selected PM2.5-bound elements
Source: PLoS One. 2024 Mar 20;19(3):e0287187. doi: 10.1371/journal.pone.0287187 (PMC10954151; doi:10.1371/journal.pone.0287187)
Supplement: S7 Table — (PDF) [file pone.0287187.s008.pdf]

S7 Table. Pearson correlation coefficients of heavy metals in $PM_{2.5}$ collected at POS.

	Na	Mg	Al	Si	S	Cl	K	Ca	Sc	Ti	V	Cr	Mn	Fe	Co	Ni	Cu	Zn	Ga	As	Se	Br	Sr	Ba
Na	1.000																							
Mg	**0.977**	1.000																						
Al	0.201	0.330	1.000																					
Si	**0.970**	**0.963**	0.135	1.000																				
S	-0.014	-0.111	-0.283	-0.162	1.000																			
Cl	0.022	-0.077	-0.096	-0.095	0.229	1.000																		
K	-0.039	-0.105	-0.162	-0.165	**0.907**	0.116	1.000																	
Ca	-0.065	-0.065	-0.216	-0.080	0.109	0.039	0.180	1.000																
Sc	0.156	0.200	0.002	0.223	-0.274	-0.115	-0.183	0.256	1.000															
Ti	0.098	0.083	-0.147	0.049	0.520	0.062	0.594	0.443	0.088	1.000														
V	-0.156	-0.163	-0.044	-0.138	-0.034	0.089	-0.072	0.098	0.126	0.084	1.000													
Cr	0.088	0.025	-0.054	0.010	0.233	0.482	0.138	-0.011	-0.151	0.015	0.204	1.000												
Mn	0.155	0.117	-0.033	0.054	0.539	0.220	0.586	0.299	-0.196	0.549	0.035	0.227	1.000											
Fe	0.110	0.050	-0.133	-0.002	**0.706**	0.181	**0.766**	0.211	-0.072	**0.701**	0.061	0.359	0.667	1.000										
Co	0.191	0.205	0.060	0.223	-0.070	-0.014	-0.028	-0.032	0.293	0.025	-0.074	-0.076	-0.140	-0.015	1.000									
Ni	-0.027	-0.079	-0.235	-0.078	0.548	0.199	0.577	-0.017	-0.029	0.551	-0.021	0.319	0.439	**0.690**	0.145	1.000								
Cu	0.104	0.086	-0.094	0.104	0.153	0.059	0.213	0.342	0.173	0.342	0.258	0.165	0.456	0.355	-0.071	0.122	1.000							
Zn	-0.110	-0.140	-0.152	-0.182	**0.690**	-0.017	**0.741**	0.062	-0.326	0.557	-0.088	-0.129	0.602	0.629	0.044	0.536	0.129	1.000						
Ga	-0.347	-0.314	0.113	-0.318	-0.144	-0.083	0.017	-0.167	-0.014	-0.116	-0.002	-0.097	-0.183	-0.033	0.115	0.043	-0.150	0.030	1.000					
As	0.073	0.022	-0.096	-0.004	0.548	0.023	0.508	0.188	-0.182	0.111	0.186	0.169	0.177	0.211	0.018	0.015	0.033	0.249	-0.103	1.000				
Se	-0.012	-0.066	-0.064	-0.088	0.612	0.268	**0.699**	0.064	-0.074	0.488	0.029	0.080	0.488	0.598	0.076	0.591	0.012	0.582	0.005	0.193	1.000			
Br	-0.111	-0.207	-0.254	-0.239	**0.898**	0.274	**0.921**	0.067	-0.293	0.531	0.001	0.231	0.530	**0.736**	0.002	0.654	0.107	**0.734**	0.043	0.470	**0.712**	1.000		
Sr	-0.166	-0.197	-0.423	-0.148	0.050	0.197	0.046	0.452	0.260	0.121	-0.027	0.017	0.037	-0.004	0.200	0.011	0.117	-0.035	-0.034	0.019	-0.056	0.090	1.000	
Ba	-0.242	-0.199	0.092	-0.259	-0.056	-0.049	-0.090	-0.031	-0.377	-0.068	-0.179	-0.125	0.029	-0.096	-0.334	-0.164	-0.243	0.195	0.028	-0.072	-0.063	-0.049	-0.244	1.000

*Please note that any value higher than 0.7 was highlighted in bold font
